# Metabolic Reprogramming of Barley in Response to Foliar Application of Dichlorinated Functional Analogues of Salicylic Acid as Priming Agents and Inducers of Plant Defence

**DOI:** 10.3390/metabo13050666

**Published:** 2023-05-17

**Authors:** Claude Y. Hamany Djande, Paul A. Steenkamp, Lizelle A. Piater, Fidele Tugizimana, Ian A. Dubery

**Affiliations:** Research Centre for Plant Metabolomics, Department of Biochemistry, University of Johannesburg, P.O. Box 524, Auckland Park, Johannesburg 2006, South Africa; claudeh@uj.ac.za (C.Y.H.D.); psteenkamp@uj.ac.za (P.A.S.); lpiater@uj.ac.za (L.A.P.); ftugizimana@uj.ac.za (F.T.)

**Keywords:** antimicrobial metabolites, barley, *Hordeum vulgare*, liquid chromatography, mass spectrometry, metabolomics, multivariate data analysis, secondary metabolites

## Abstract

Designing innovative biological crop protection strategies to stimulate natural plant immunity is motivated by the growing need for eco-friendly alternatives to conventional biocidal agrochemicals. Salicylic acid (SA) and analogues are known chemical inducers of priming plant immunity against environmental stresses. The aim of the study was to study the metabolic reprogramming in barley plants following an application of three proposed dichlorinated inducers of acquired resistance. 3,5-Dichloroanthranilic acid, 2,6-dichloropyridine-4-carboxylic acid, and 3,5-dichlorosalicylic acid were applied to barley at the third leaf stage of development and harvested at 12, 24, and 36 h post-treatment. Metabolites were extracted using methanol for untargeted metabolomics analyses. Samples were analysed by ultra-high performance liquid chromatography coupled to high-definition mass spectrometry (UHPLC-HDMS). Chemometric methods and bioinformatics tools were used to mine and interpret the generated data. Alterations in the levels of both primary and secondary metabolites were observed. The accumulation of barley-specific metabolites, hordatines, and precursors was observed from 24 h post-treatment. The phenylpropanoid pathway, a marker of induced resistance, was identified among the key mechanisms activated by the treatment with the three inducers. No salicylic acid or SA derivatives were annotated as signatory biomarkers; instead, jasmonic acid precursors and derivatives were found as discriminatory metabolites across treatments. The study highlights differences and similarities in the metabolomes of barley after treatment with the three inducers and points to the triggering chemical changes associated with defence and resistance. This report is the first of its kind, and the knowledge acquired provides deeper insight into the role of dichlorinated small molecules as inducers of plant immunity and can be used in metabolomics-guided plant improvement programmes.

## 1. Introduction

Plants are naturally exposed to a plethora of stresses, which severely affect their growth and yield. Upon exposure to biotic stresses, molecular communication with the plant takes place and may lead to a beneficial association or a disease. In this interaction between two biological organisms, the resistance or susceptibility is determined by the ability to ward off/defend against the stress-inducing environment in which each finds itself. Plants have evolved an innate immune system against microbial threats consisting of preformed defensive barriers and constitutive chemical deterrents and inducible defences based on newly synthesised defence proteins and antimicrobial chemicals [1,2,3]. Resistance responses might not always be effective due to not being timeously triggered or not being launched at the required intensity. Moreover, some pathogens have developed certain strategies to circumvent host defences, thus resulting in disease. Abiotic environmental stressors can also have a negative impact on the resistance or tolerance of plants towards a specific strain of pathogen [4], necessitating the use of antimicrobial chemicals for disease control. As a result of public concern over the risks associated with pesticide usage, alternative control strategies are receiving much attention in order to limit unfavourable hazardous environmental side effects.

Induced resistance (IR) can be described as an altered metabolic state that is activated once the host plant’s immune system is triggered or activated by a pathogen attack or other biotic stresses [5]. Fundamentally, as part of IR, systemic acquired resistance (SAR) is known to depend on salicylic acid (SA) and induced systemic resistance (ISR) relies on beneficial microbes and hormones, such as jasmonic acid (JA) and ethylene (ET). However, there is an interconnection involving more participatory mechanisms than SAR and ISR in IR, creating a multi-layered network defining plant immunity [6,7,8,9]. The mediation of SAR by the accumulation of SA has previously been demonstrated [5,10,11,12,13,14]. SAR is often effective against a wide spectrum of pathogens and is regarded as the most agronomically important type of plant immunity [15]. The activation of plant immune mechanisms can be initiated by (a non-virulent) pathogen attack but also by treatment with natural or synthetic compounds, such as SA and synthetic analogues [16,17]. Studies on IR elicited by chemicals have revealed previously unknown features of the plant defence response, including defence priming [18].

In addition to the mentioned IR associated with innate immunity, plants can also acquire immunity upon treatment with certain biotic and abiotic stimuli, a phenomenon mediated by preconditioning or priming for inducible defence [19,20]. Immune priming or potentiation enables faster and/or stronger induction of inducible defences following a successful attack by pathogens. As priming events boost multigenic basal resistance, the ensuing protection against disease can be more resilient than race-specific resistance, which is based on single resistance genes. Regardless of the fact that priming rarely provides complete disease protection [21], the application of priming-inducing agents is increasingly considered for exploitation in integrated pest and disease management [22,23].

SA performs a crucial role as an endogenous signalling molecule that activates various aspects of plant defence. The phytohormone also performs important roles in growth and development, respiratory pathways, and regulation of redox homeostasis [24,25,26]. The exogenous application of SA and related synthetic analogues activating the plant defence system has been reported in several studies [27,28,29,30,31]. Although very efficient in inducing plant resistance, the phytotoxicity and rapid glycosylation of SA result in reduced efficacy and have prevented its use as a plant protection molecule. Hence, synthetic chemical analogues of SA, capable of mimicking various functions thereof, represent an attractive alternative/substitute to the use of conventional biocidal agrochemicals [23,32]. Substitution of SA with the electron-withdrawing element chlorine has displayed an increase in SA activity [33]. Mono- and dichloro-substituted SA derivatives, such as 3,5-DCSA and 2,6-dichloroisonicotinic acid (2,6-DCINA), were found to successfully increase tobacco plant resistance to pathogen attack through the induction and accumulation of pathogenesis-related (PR) proteins [31,34]. It was further established that mono or multiple substitution(s) at positions three and/or five on SA were more active at inducing the PR-1a protein than those at positions two, four, and six, and SA itself [33]. Relatedly, a screening of 60,000 unique chemicals for inducers of pathogen-responsive reporter genes in Arabidopsis seedlings led to the discovery of 114 synthetic elicitor candidates, including 3,5-dichloroanthranilic acid (3,5-DCAA). Upon treatment with 3,5-DCAA, Arabidopsis effectively develops resistance to virulent isolates of the oomycete *Hyaloperonospora parasitica* and *Pseudomonas syringae* DC3000 [28].

Adaptive metabolic reprogramming in plants is often a result of the exposure to a single or a multitude of external stimuli or environmental pressures. Such variation can be observed in a wide variety of primary and secondary metabolites, including ionic inorganic compounds, hydrophilic carbohydrates, amino acids, organic compounds, and compounds linked to hydrophobic lipids. Investigating the metabolic perturbations caused by chemical activators of priming or inducers of IR can significantly contribute to gaining insights into the unique vs. shared features of the host response towards these inducers. In this study, the host was barley (*Hordeum vulgare* L., ‘Hessekwa’ cultivar), the fourth most important cereal crop in the world. Barley is primarily used for animal feed and human food and alcoholic beverage production. The widely adaptable, short-seasoned, and early maturing crop is farmed as a summer or winter crop in temperate and tropical climates, respectively. In plant science, barley is commonly used as a model plant, particularly when assessing plant resilience to environmental stress [35,36].

Untargeted metabolomics approaches combined with advanced chemometric tools were employed to investigate the metabolic reprogramming in leaves following treatment with synthetic (functional) analogues of SA: 3,5-dichlorosalicylic acid (3,5-DCSA), 3,5-dichloroanthranilic acid (3,5-DCAA), and 2,6-dichloropyridine-4-carboxylic acid (2,6-DCP-4-CA, also known as 2,6-DCINA), in order to evaluate their potential for the induction of a state of enhanced resistance in barley plants. The importance of metabolic reprogramming and priming to improve abiotic stress tolerance in a variety of significant crops is being supported by growing research.

## 2. Materials and Methods

### 2.1. Barley Plant Material and Growth Conditions

Barley seeds were collected from the experimental line ‘Hessekwa’ cultivated in Bredasdorp, in the Western Cape region of South Africa. ‘Hessekwa’ is a rainfed winter crop, and seeds were provided by the South African Barley Breeding Institute (SABBI). In the current study, seeds were grown as previously described [37,38]. Briefly, the plants were grown in a plant growth room under well-controlled conditions: 12 h fluorescence light (≈85 μmol m^−2^ s^−2^) and 12 h dark cycle at 22–27 °C. Surfaced-sterilised (70% ethanol) and soaked (in sterile water for 2 h) barley seeds were planted in pasteurised (at 70 °C) soil. The plants were watered twice a week with distilled water containing a water-soluble chemical fertiliser (Multisol ‘N’, Culterra, Muldersdrift, South Africa). The plants were grown for 21 d (or 16 d post-emergence) before treatment with the inducers. At that time, the seedlings were at the physiological stage 13 according to the Zadoks growth and development scale [37,38].

### 2.2. Barley Plant Treatment with Priming Inducers

All chemicals, namely, 3,5-dichlorosalicylic acid (3,5-DCSA), 2,6-dichloropyridine-4-carboxylic acid (2,6-DCP-4-CA), and 3,5-dichloroanthranilic acid (3,5-DCAA) were obtained from Merck–Sigma-Aldrich, Johannesburg, South Africa. These inducers were dissolved in dimethylsulphoxide (DMSO, 1 μL/mL; BDH Chemicals, England) mixed with 0.05% of wetting agent (Effekto, Pretoria, South Africa). Control plants received the same concentration of DMSO and wetting agent. Following the foliar application (40 sprays = ±6 mL/pot) of 200 μM of 3,5-DCSA, 2,6-DCP-4-CA, and 3,5-DCAA, treatment groups were kept separately, and leaves (the entire aerial parts of the plants, 1 cm above the surface) were harvested after 12, 24, and 36 h, respectively. The concentration of the inducers and the time interval of the investigation were chosen based on several optimisation studies that indicated no morphological effects. The experimental design included three independent biological replicates for each treatment.

### 2.3. Metabolite Extraction from Seedlings and Pre-Analytical Sample Preparation

Barley shoot tissues were harvested and snap-frozen in the liquid nitrogen to quench metabolic activity. Metabolites were extracted from the leaves of each replicate with 80% cold aqueous methanol (1:10 *w*/*v* ratio). Following homogenisation for 1 min with an Ultra-Turrax homogeniser, and sonication for 10 s with a probe sonicator (Bandelin Sonopuls, Berlin, Germany), the homogenates were centrifuged at 5100× *g* and 4 °C for 20 min. Supernatants were concentrated to 1 mL using a rotary evaporator at 45 °C and further evaporated to complete dryness in a dry bath pre-heated at 45 °C. The reconstitution of dried extract was completed with 50% UHPLC-grade methanol (Romil, Cambridge, UK) in a 1:10 *m*/*v* ratio. In preparation for chromatographic analyses, extracts were filtered through 0.22 μm nylon filters into chromatography vials fitted with 500 μL inserts, capped, and stored at 4 °C.

### 2.4. Sample Analyses on Mass Spectrometry-Based Analytical Platforms (Ultra-High Performance Liquid Chromatography—High Definition Mass Spectrometry (UHPLC-HDMS))

The Waters Acquity UHPLC hyphenated with a Waters SYNAPT G1 QTOF (quadrupole time-of-flight) mass spectrometer system (Waters Corporation, Milford, MA, USA) was used to analyse the aqueous-methanol extracts. The chromatographic separation of samples was completed using a Waters HSS T3 reverse phase C18 column (150 mm × 2.1 mm × 1.8 μm) thermostatted at 60 °C. The concave gradient elution was carried out at a flow rate of 0.4 mL.min^−1^ using a binary solvent system consisting of water (eluent A) and acetonitrile (eluent B; Romil Pure Chemistry, Cambridge, UK), both of which contained 0.1% formic acid. For the first min of the elution, 95% A and 5% B were kept constant. When the gradient was applied, the chromatographic condition was changed to 10% A and 90% B for 10 s, followed by 5% A and 95% B for 1 min and 50 s, before being restored to the original condition at the end of 28 min. Each sample was injected with a 2 μL volume over the course of a 30 min run. To prevent measurement bias, all sample extracts were randomised. Additionally, pooled quality control (QC) samples were also used to evaluate the stability of the LC-MS system. Blanks consisting of 50% MeOH were used to monitor potential carry-over. Data acquisition was based on three independent biological replicates, and each was analysed in triplicate; thus, *n* = 9.

The TOF MS analyser was used in V-optics mode, and centroid spectral data were acquired using both positive and negative electrospray ionisation (ESI), with a scan range of 50–1200 Da and a scan time of 0.1 s. The cone and desolvation gas flows were at 50 L.h^−1^ and 550 L.h^−1^, respectively. Nitrogen was used as a nebuliser gas at a flow rate of 700 50 L.h^−1^. The sampling and extraction cone voltages were 40 V and 4.0 V, respectively, while the capillary voltage was set at 2.5 kV. The desolvation temperature was set at 450 °C, and the source temperature was fixed at 120 °C. Leucine encephalin (50 pg·mL^−1^, [M + H]^+^ = 556.2771 and [M − H]^−^ = 554.2615) was used as lock mass, sampled every 15 s, and producing an average intensity of 350 counts per scan. The lock mass serves to correct the centroid mass values in the sample for small deviations from the accurate mass measurement. Both unfragmented and fragmented (using an MS^E^ method, 10–40 eV) data were acquired. Fragmentation data were used for downstream metabolite structural elucidation and annotation.

### 2.5. Chemometrics: Data Mining

UHPLC-MS extracted raw data (analysed in positive and negative ionisation modes) was processed using the MarkerLynx XS™ application management tool of the MassLynx XS software, version 4.1 (Waters Corporation, Milford, MA, USA) to generate the corresponding data matrices. For precise peak detection and alignment, the software uses the unique *ApexTrack* (also termed *ApexPeakTrack*) algorithm. A modified Savitzky-Golay smoothing and integration was used prior to the computation of peak intensities. The sample was normalised using the total ion intensities associated with each peak. The processing parameters were set at a mass range of 50–1200 Da, a mass window of 0.05 Da, the intensity threshold (noise elimination level parameter) was set at 100 counts, a mass tolerance of 0.05 Da, and a retention time (Rt) range of 1.5–25.0 min of the chromatograms.

For statistical modelling, generated data matrices were exported to the ‘soft independent modelling of class analogy’ (SIMCA) software, version 14, equipped with the ‘omics skin’ function (Sartorius, Umeå, Sweden). As specified in the results section, data were scaled prior to computing chemometric models. As part of the chemometrics models generated, the unsupervised method, principal component analysis (PCA), was applied to reduce the dimensionality of the data and to reveal groupings, trends, and similarities existing between treatments. Moreover, the supervised model, orthogonal projection to latent structures-discriminant analysis (OPLS-DA), was also computed to classify the samples (binary classification), generate descriptive statistics, and provide potential biomarkers. Models were evaluated using the predictive power, Q^2^, and the explained cumulative variation in the matrix X, R^2^X (cum), also known as the ‘goodness of fit’ parameter. Additionally, the cross-validated predictive residual analysis of variance (CV-ANOVA) was taken into account to statistically evaluate the accuracy of the OPLS-DA models created. A *p*-value of less than 0.05 suggested a strong model. Moreover, a permutation test was performed on 100 randomly initiated permutations to validate the models. Discriminant features or ions, with both high correlation and covariation, are located at the extreme ends of the generated loading S-plots (e.g., [p(corr) ≥ 0.5, ≤−0.5, and (p1) ≥ 0.1, ≤−0.1]) and their statistical significance was assessed on variable importance in projection (VIP) plots which summarise the importance of features. The VIP scores determine the level of significance of each ion in the dataset, and all selected features had VIP scores between 1 and 2.

In the MetaboAnalyst 5.0 (www.metaboanalyst.ca/, accessed on 1 May 2022) [39] platform, dendrogram heatmaps, which allowed the visualisation of the distribution of selected metabolites across the conditions (different time-points and treatments), were constructed. In addition, Metabolomics Pathway Analysis (MetPA), also housed in the MetaboAnalyst bioinformatics software, was used to uncover the key metabolic pathways that define induced responses in barley treated with 3,5-DCAA, 2,6-DCP-4-CA, and 3,5-DCSA. KEGG (Kyoto Encyclopedia of Genes and Genomes, www.genome.jp/kegg/pathway.html, accessed on 10 May 2022) [40] identifiers for each annotated metabolite were used as inputs, and high-quality KEGG metabolic pathways were used as the backend knowledge base.

### 2.6. Metabolite Annotation

To comprehensively annotate the measured metabolome of barley shoot tissue, molecular networking methods were applied. Briefly, following conversion of ‘Waters’ (.raw) spectral files into the ‘analysis base file’ (ABF) format using Reifys Abf converter software [41], the spectral data were uploaded into the Mass Spectrometry-Data Independent AnaLysis (MS-DIAL; http://prime.psc.riken.jp/, accessed on 15 May 2022) [42] software for processing with parameters set at mass accuracy 0.05; minimum peak height 10 amplitude, and the Rt tolerance 0.2 min. The MS-DIAL processed files (GnpsMgf and GnpsTable) were then exported into the Global Natural Product Social Sphere (GNPS; https://gnps.ucsd.edu/, accessed on 3 June 2022) [43] using the WinSCP server for molecular networking. A feature-based molecular networking (FBMN) was computed with a mass tolerance set at 0.05; the minimum pair cosine score was set at 0.6 with a minimum of 4. The search for analogues setting was turned off. Furthermore, to increase the chemical insights that can be obtained from the spectral data, FBMN outputs were combined with outputs from substructural annotation by MS2 latent Dirichlet allocation (MS2LDA), in silico annotation (by Network Annotation Propagation, NAP), and the automated chemical classification (through ClassyFire) into the enhanced molecular networking workflow, MolNetEnhancer. MS2LDA is a tool that decomposes molecular fragmentation data derived from large metabolomics experiments into annotated Mass2Motifs or discovers Mass2Motifs from experimental data, while MolNetEnhancer enables the chemical annotation, visualisation, and discovery of the subtle substructure diversity within molecular families [44,45]. Furthermore, an expert-guided metabolite annotation was performed by manually inspecting individual spectra and mass fragmentation as previously described [37,38]. The metabolite annotation and putative identification was completed and confidently reported herein in accordance with level 2 of the Metabolomics Standards Initiative (MSI) [46].

## 3. Results

### 3.1. Chromatographic and Mass Spectrometric Analyses and Molecular Networking Approach to Uncover the Metabolic Space of Barley Leaves Treated with Inducers

**Figure 1:**
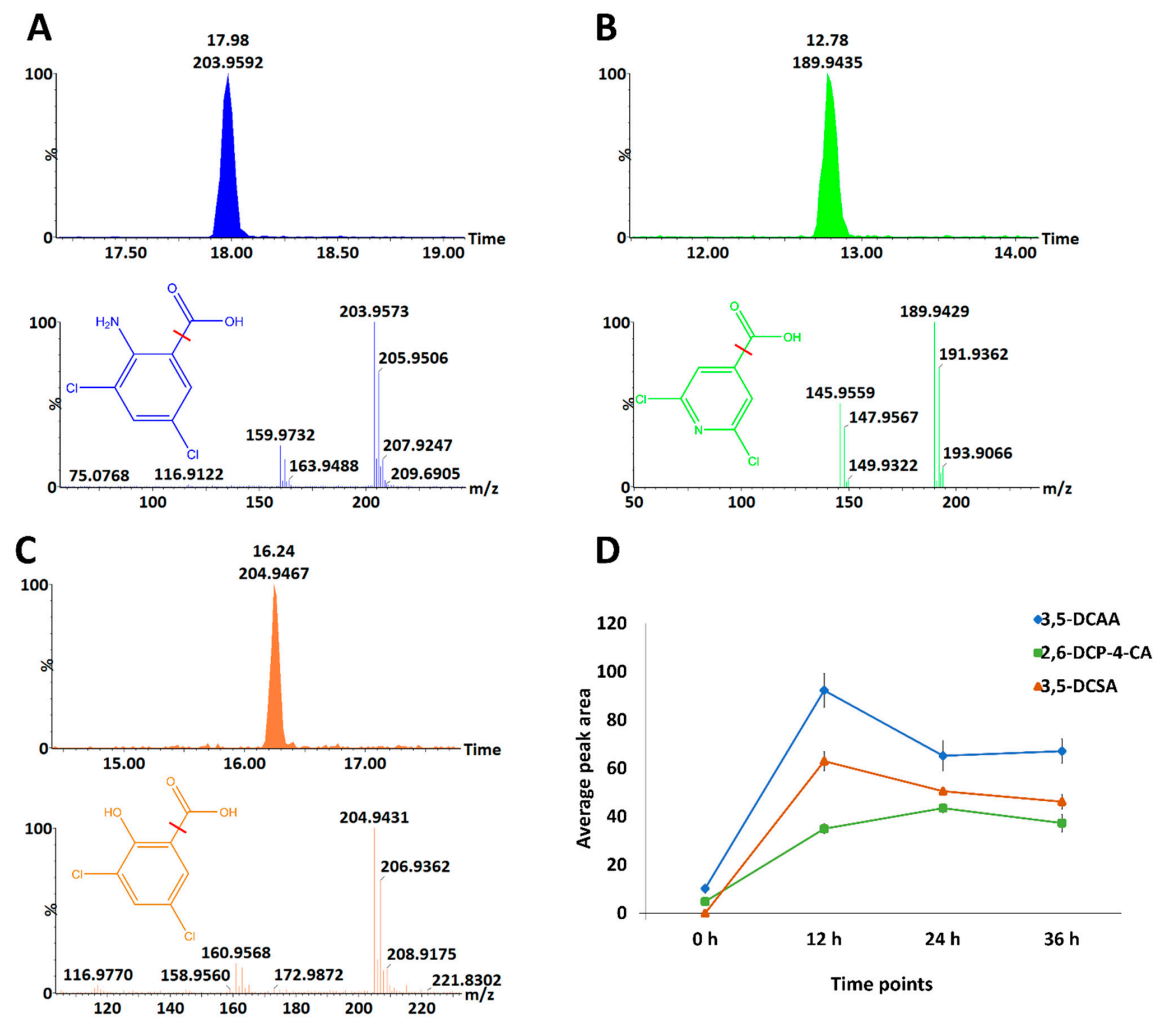
Presence of xenobiotic dichlorinated inducers in barley shoot tissue extracts from different time points. (**A**–**C**): Extracted ion chromatograms (XIC) and corresponding mass spectrum characteristics of the inducers: 3,5-DCAA, 2,6-DCP-4-CA, and 3,5-DCSA; the structure of each compound is also represented, and the fragmentation site is indicated with the red line. (**D**): Relative concentration of inducers in barley shoot tissue. Error bars indicate the standard deviations of the average peak areas of the samples.

Metabolomics studies aim to identify and quantify small molecules involved in metabolic processes. Due to its high throughput and good coverage of metabolites, UHPLC–MS has increasingly been used as a platform of choice for such studies when combined with soft electrospray ionisation (ESI). Reported herein, reverse phase chromatographic separation with mass spectrometric detection revealed differences in peak population (presence or absence) and peak intensities (reduced or increased) of analytes present in methanol-extracted samples, visible on the MS chromatograms. These are time- and treatment-related differences observed between the controls (non-treated) and treated conditions (Figures S1–S3). In addition, a broad coverage of midpolar to nonpolar was also noted with analytes eluting throughout the run time. It is worth pointing out that all three inducers, 3,5-DCAA, 3,5-DCSA, and 2,6-DCP-4-CA, were detected in the samples from treated plants. These inducers were characterised as depicted in Figure 1A–C, showing the extracted ion chromatograms (XIC) of each inducer and the corresponding spectra and structures. A pattern representative of the presence of chlorine atom(s) was observed. The two isotopes of chlorine (^35^Cl and ^37^Cl) displayed fragment ions separated by two *m*/*z* units. In addition, the inducers were also characterised by the loss of the carboxyl group (neutral loss, NL:45) generating fragments with *m*/*z* of 159/161, 145/147, and 160/162 for 3,5-DCAA, 2,6-DCP-4-CA, and 3,5-DCSA, respectively. In some cases, the loss of an HCl (NL:36) molecule was also observed. Furthermore, the presence of inducers or their absorption by barley shoot tissue was demonstrated by an increase in the relative concentration (peak area intensities) over time before dropping to the point of stability. The highest relative concentration in barley was observed at 12 h for 3,5-DCAA and 3,5-DCSA; and at 24 h for 2,6-DCP-4-CA. 3,5-DCAA was the highest in the plants at all the time points under investigation (12 h, 24 h, and 36 h) (Figure 1D).

**Figure 2:**
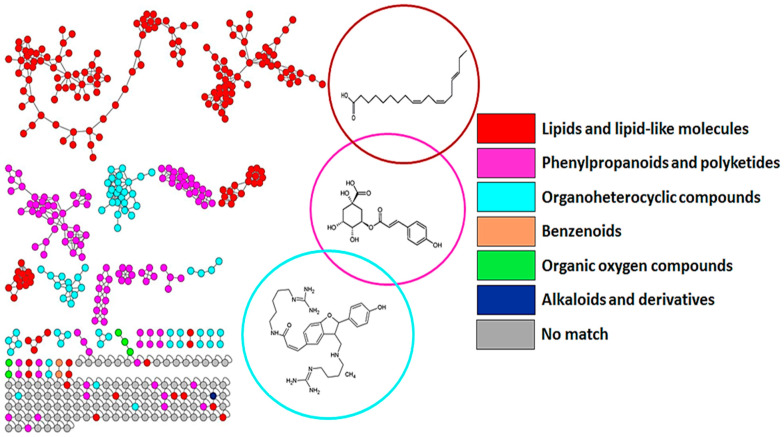
MolNetEnhancer network of ESI(–) spectra obtained from shoot extracts of the barley (‘Hessekwa’) samples. The enriched molecular network depicts structurally similar nodes as molecular families/clusters, with the annotated metabolites, MS2LDA (MS2 latent Dirichlet allocation) substructures, and Network Annotation Propagation (NAP) annotations, assigned class annotations, represented by coloured nodes and nodes with no class annotation as grey.

For metabolome characterisation, a molecular networking (MN) strategy, i.e., MolNetEnhancer, was applied (as described in the methodology section). This allowed a comprehensive exploration and enrichment of chemical annotations, discovering the subtle substructural diversity within molecular families. Furthermore, this MN strategy allowed the visualisation of molecular families with class annotations. Six molecular families were visualised and highlighted in Figure 2. These were lipids and lipid-like molecules, phenylpropanoids and polyketides, organoheterocyclic compounds, benzenoids, organic oxygen compounds, and alkaloids and derivatives. When zooming into these superfamilies, the phenylpropanoids and polyketide superfamily was dominated mainly by flavonoid glycosides. These superfamilies of metabolites served as a template for the annotation of the discriminant metabolites.

### 3.2. Multivariate Data Analyses: Statistical Description, Evaluation, and Exploration of Changes Observed in the UHPLC-MS Data

**Figure 3:**
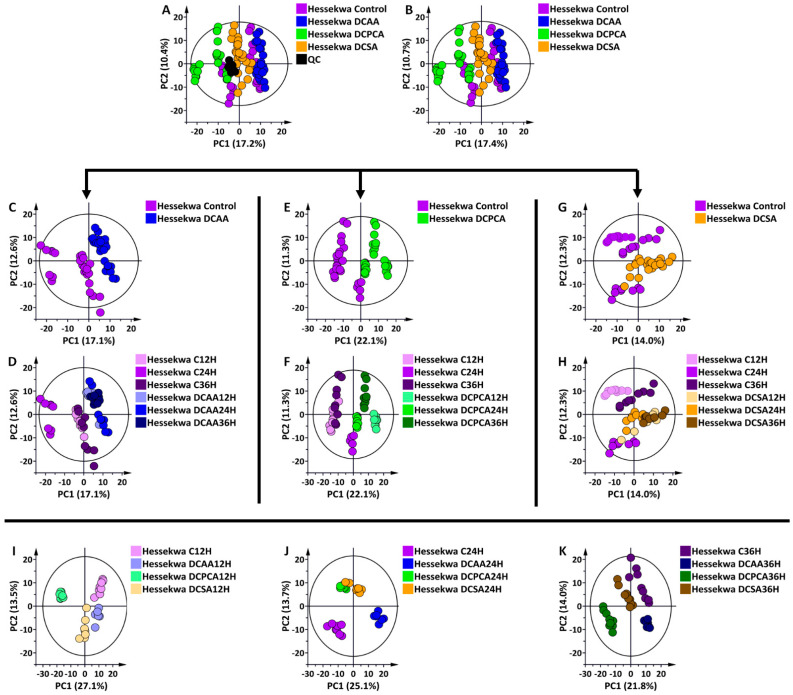
Principal component analysis (PCA) score plots of ESI(–) data from shoot extracts of the ’Hessekwa’ cultivar of *Hordeum vulgare*. All data were UV (unit variance) scaled, and the calculated Hoteling’s T^2^ with a 95% confidence interval is represented by the ellipses present in each PCA score plot. (**A**): a 12-component model of all conditions (including quality controls, QCs), explaining 59.5% variation and predicting 41.6% variation; (**B**) a 12-component model of all conditions (excluding QCs) explaining 60.7% variation, and predicting 42.2% variation. (**C**): a 6-component model of 3,5-DCAA treated and non-treated samples at 12, 24, and 36 h, respectively, explaining 54.6% variation and predicting 37.2% variation; (**D**): same as (**C**) but coloured based on time points; (**E**): a 7-component model of 2,6-DCPCA treated and non-treated samples, explaining 60.6% variation and predicting 41.1% variation; (**F**): same as (**E**) but coloured based on time points. (**G**): a 7-component model of 3,5-DCSA treated and non-treated sample, explaining 53.8% variation and predicting 29.9% variation; (**H**): same as (**G**) but coloured based on time points. (**I**): a 4-component model of all treated and non-treated samples at 12 h, explaining 56.5% variation, and predicting 41.7% variation; (**J**): a 4-component model of all treated and non-treated samples at 24 h, explaining 54.4% variation, and predicting 35.5% variation; (**K**): a 4-component model of all treated and non-treated samples at 36 h, explaining 50.1% variation, and predicting 33.2% variation. (The corresponding set of diagrams for ESI (+) data is presented in Figure S4). As observed in (**A**,**B**), (**C**–**H**) were generated to better visualise the time-dependent changes in each treatment, while (**I**–**K**) highlight the treatment-dependent variation at each selected time point.

Unsupervised chemometrics methods were applied to reveal trends in the datasets obtained from the UHPL-MS analyses. The principal components analysis (PCA) allowed us to summarise information in multidimensional datasets. From the PCA models, some distinct clustering is revealed, indicating treatment- and time-related differences between and within samples (Figure 3 and Figure S4). As a general rule, samples grouped together have more in common (at a metabolome level) than those further apart.

Thus, assessing the scores plots of generated PCA models (Figure 3 and Figure S4), treatment- and time-related sample groupings can be observed, which point to underlying metabolic reprogramming within barley shoot tissue due to the inducer treatments. Except for 3,5-DCSA in the positive ESI mode, controls are separated from the treated, irrespective of the time point. Looking at the controls in each treatment, the time points 12 h and 36 h grouped together and separated from the 24 h, and a slightly similar pattern can also be observed within treated samples (Figure 3C–H). This gives an indication of a possible diurnal effect on the plants. When focusing on each time point (Figure 3I–K), a clear grouping pattern was particularly observed at 24 h between all treatments, showing 3,5-DCSA- and 2,6-DCP-4-CA-treated samples grouped but separated from the control and DCAA treated samples grouping away from the control and other treatments (Figure 3J). This observation drew more attention to the 3,5-DCAA treatment at 24 h. Although able to indicate differences between treatment groups, unsupervised chemometrics are not classification or discrimination methods *per se*, hence less informative in terms of features that explain the differences between sample groups. A supervised method was then used, as described below, to uncover features or metabolites responsible for differential sample groupings.

### 3.3. Discriminant Analyses: Treatment- and Time-Related Metabolites and Fold Changes

**Figure 4:**
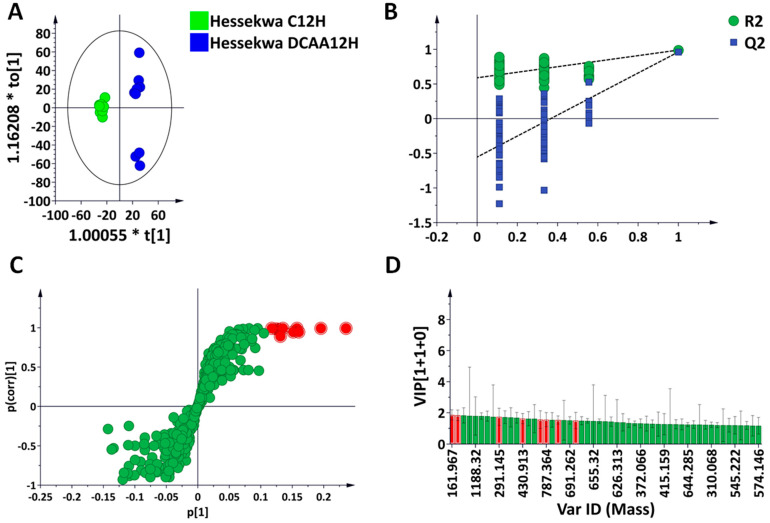
Supervised learning methods for analyses of ultra-high performance liquid chromatography–mass spectrometry (UHPLC–MS) data. Orthogonal partial least squares discriminant analysis (OPLS-DA) modelling and feature selection were performed based on the unique metabolite profiles of ‘Hessekwa C12h’ (control at 12 h) and ‘Hessekwa DCAA 12 h’ (treatment at 12 h) leaf extracts in negative ionisation mode. (**A**) OPLS-DA score plots show a clear separation between the two conditions. The model is made of one predictive component and one orthogonal component (R^2^X = 57.5%, R^2^Y = 98.9%, Q^2^ = 96.1%, CV-ANOVA *p*-value= 4.6539 × 10^−9^). (**B**) OPLS-DA validation: Permutation test analysis performed with 100 randomly selected models and showing the above model to be the best among the permutated ones. (**C**) Loading S-plots showing the features responsible for sample clustering, located at the ‘outlier’ ends of the S-plots with both high correlation and covariation, [p(corr) ≥ 0.75 and (p1) ≥ 0.1], are highlighted in red. These features are statistically significant candidates as biomarkers related to the DCAA treatment. (**D**) A variable importance in projection (VIP) plot corresponding to the model above and pointing out the mathematical significance of each feature responsible for the discrimination of the treated vs. control conditions. A VIP score >1 is considered as significant in the projection, and the higher the score values, the more significant the features are.

The supervised learning method, orthogonal projection to latent structure discriminant analysis (OPLS-DA), was performed, and an example is shown in Figure 4. In Figure 4A, the OPLS-DA score plot shows group separation in an OPLS-DA score space. The predictive capability of the computed OPLS-DA model was validated using a permutation test. This consists of comparing the observed R^2^ and Q^2^ values with randomly permutated ones (n = 100). As seen in the example below, the R^2^ and Q^2^ values of all computed models were statistically better (or higher) than the 100 permutated ones (Figure 4B). The OPLS loading S-plot allowed the extraction of variables related to each treatment and time point (Figure 4C). Features with both high correlation and covariation were considered, [p(corr) ≥ 0.5, ≤−0.5, and (p1) ≥ 0.1, ≤−0.1]. In addition, variable importance in projection (VIP) score plots were used to evaluate the statistical significance of each feature. All selected features had a variable VIP score above 1 (Figure 4D).

In total, 66 discriminant metabolites belonging to the above-mentioned superfamilies (Figure 2) were annotated/putatively identified as previously described by us [37,38]. These compounds and the corresponding fold changes are presented in Table 1. Compounds with a fold change (FC) > 1 were considered positively correlated to the treatment, and those <1 were negatively correlated. In 3,5-DCAA-treated samples, the highest fold changes were observed with isorhamnetin-3-O-glucoside (FC: 3.49) at 24 h, 4-O-*p*-coumaroylquinic (FC: 5.58), sinapoylagmatine (FC: 3.08), and isovitexin 2″-O-arabinoside (FC: 6.34) at 36 h. In the case of the 2,6-DCPCA treatment, the most positively correlated compounds were mainly fatty acids and derivatives, such as 9,12,13-trihydroxy-10,15-octadecadienoic acid (9,12,13-triHODE) isomer II (FC: 10.73), trihydroxyoctadecenoic acid (triHOME) (FC: 4.59) at 12 h; 9,12,13-triHODE isomer I, 9-oxo-12,13-dihydroxy-10E,15Z-octadecadienoic acid (9-oxo-12,13-diHODE), and a 12-oxophytodienoic acid (OPDA) conjugate and linolenoylglycerol isomer I, at both 12 and 36 h. With the 3,5-DCSA treatment, hordatine A isomer II (FC: 3.49), isovitexin 2″-O-glucoside (FC: 10.97), isovitexin 2″-O-arabinoside (FC: 11.45), and a proline (Pro) derivative, Pro betaine (FC: 15.26), were metabolites with the highest fold change at 36 h. It was also noted that except for valine and proline betaine in extracts from 3,5-DCSA-treated leaves, all amino acids were negatively correlated to all treatments. Subsequently, Venn diagrams were generated for better visualisation of differences across time in individual treatments and at each time point and across treatments.

### 3.4. Time-Related Differences and Similarities in the Chemical Profiles of Barley Leaves, following Foliar Application of 3,5-DCAA, 2,6-DCP-4-CA and 3,5-DCSA

**Figure 5:**
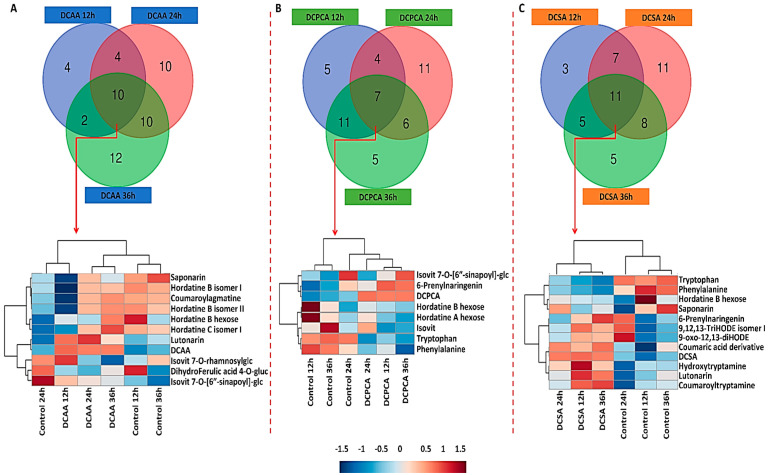
Venn diagrams and dendrogram heatmaps of discriminant metabolites. The distribution of metabolites across time points (12, 24, and 36 h) following foliar application of (**A**) 3,5-DCAA, (**B**) 2,6-DCP-4-CA, and (**C**) 3,5-DCSA is displayed. While Venn diagrams were generated from all the annotated discriminant metabolites (Table 1), the dendrogram heatmaps were generated from shared metabolites (red arrows) across all three-time points to highlight the differences in the relative concentration over time. Metabolites common to the two groups are discussed in the text. The numerical values in the Venn diagram correspond to the number of unique metabolites at each time-point and overlapping metabolites across the different conditions.

The Venn diagrams in Figure 5 indicate that there is a wide diversity of metabolites distributed across time in each treatment condition. Starting with extracts from 3,5-DCAA-treated leaves (Figure 5A; Table 1), four metabolites were specifically found as discriminant at 12 h, and these were feruloylagmatine (a precursor in the biosynthesis of hordatines B and C), an isomer of hordatine A, linolenoylglycerol, and the flavonoid apigenin 6-C-arabinoside 8-C-glucoside. At 24 h, specific discriminant metabolites included organic acids citric and isocitric acid, flavonoids, such as isoscoparin 2″,6″-di-O-glucoside, and phenolic- and fatty-derivatives. Twelve discriminant metabolites were found specifically at 36 h post-treatment with 3,5-DCAA. These included some amino acids, a chlorogenic acid and sinapoylagmatine. As part of the flavonoids and fatty acid derivatives, isovitexin 7,6″-di-O-glucoside, apigenin 7-O-arabinosylglucoside, 12-hydroxyjasmonate sulphate, and linolenoylglycerol isomers I and II were noted. In addition to the specific metabolites annotated, some were overlapping across time points. In total, 10 signatory metabolites were found across all time points and were represented on a dendrogram heatmap to show their differential distribution and to highlight the relationship among the sample groups. As seen on the dendrogram heatmap below the Venn diagram in Figure 5A, the common metabolites were mainly the barley-specific hydroxycinnamic acid amides (HCAAs), hordatine B isomer I and II, and the corresponding glycosylated form, hordatine C isomer I, and coumaroylagmatine. In addition, dihydroferulic acid 4-O-glucuronide, lutonarin (luteolin as aglycone), saponarin, isovitexin 7-O-rhamnosylglucoside, and isovitexin 7-O-[6″-sinapoyl]-glucoside (all with apigenin as aglycone) were also found. The heatmap shows an increase in the relative concentration of the HCAAs over time. Lutonarin was also up-regulated at each time point. The dendrogram corresponding to the samples shows treatments branching apart from controls and an interesting association of control samples at 12 and 36 h, both separated from 24 h.

Regarding extracts from 2,6-DCP-4-CA-treated leaves, five metabolites of the phenylpropanoids and fatty acid derivatives were found exclusively at 12 h. At 24 h, 11 metabolites were specific to the time point, amongst which were 3-O-*p*-coumaroylquinic acid, coumaroylputrescine, sinapoylagmatine, a feruloyagmatine derivative, coumaroylhydroxyagmatine, 3-hydroxycoumarin, and hydroxytryptamine. Finally, at 36 h, the five specific discriminant metabolites included feruloylagmatine, hordatine A isomer II, dihydrojasmonic acid conjugate, and linolenoylglycerol isomers II and VI. Four metabolites were overlapping across the 12 and 24 h time points and between 12 and 36 h, where 11 metabolites were shared and included coumaroyltryptamine, sinapoylhydroxyagmatine, saponarin, Tyr, and linolenoylglycerol isomer I, to list a few. Coumaroylagmatine, lutonarin, and malic acid were among the six metabolites shared between 24 and 36 h. Seven discriminant metabolites were overlapping across all time points. Looking at the dendrogram heatmap in Figure 5B, hordatine A and B hexose were less abundant at 12 h and 36 h. At these same time points, isovitexin 7-O-[6″-sinapoyl]-glucoside and 6-prenylnaringenin were more abundant. Isovitexin followed the same pattern as hordatine A and B hexose and was more abundant at 24 h. A down-regulation of Trp and Phe at all-time points was also noted. On the dendrogram, the ‘treatment’ groups were clearly separated from the corresponding controls; however, the treatment at 24 h was closer to the corresponding control.

In extracts from 3,5-DCSA-treated leaves, three metabolites were specifically found at 12 h, namely, hordatine A hexose, isovitexin 7-O-[X″-feruloyl]-glucoside and Val. At 24 h, 11 metabolites were found, specific among which were hordatine B and hordatine C, and asparaginylglucose. Finally, five were found at 36 h and included hordatine A isomer II, isovitexin 7,6″-di-O-glucoside, apigenin 7-O-arabinosylglucoside, dihydrojasmonic acid, and proline betaine. Here, seven metabolites were shared among the 12 and 24 h treatment groups, including hordatine C hexose isomer I, malic acid, and an oxylipin, 12-oxo-phytodienoic acid (OPDA) conjugate. Sinapic acid hexose, linolenoylgycerol isomer I and II, Tyr, and Ile were overlapping between 12 and 36 h. Finally, coumaroylagmatine, coumaroylhydroxyagmatine, sinapoylhydroxyagmatine, hordatine A, and C isomer I was among the eight metabolites shared between 24 and 36 h. Common metabolites to all three-time points included Trp and Phe, both down-regulated at all time points and lutonarin, hydroxytryptamine and coumaroyltryptamine, all up-regulated throughout, as shown on the heatmap. Again, all control samples clustered separately from the treated and control 24 h branches away from control 12 and 36 h, respectively (Figure 5C).

### 3.5. Treatment-Related Similarities and Differences in the Chemical Profile of Barley Leaves, following Foliar Application of 3,5-DCAA, 2,6-DCP-4-CA, and 3,5-DCSA

**Figure 6:**
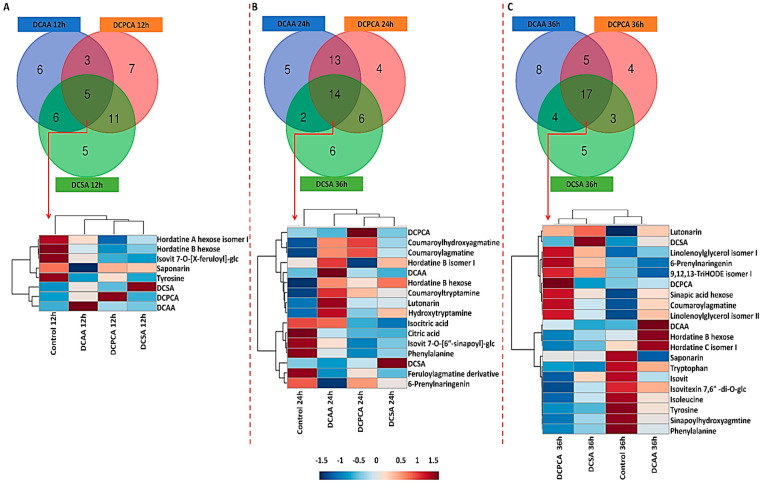
Venn diagrams and dendrogram heatmaps of discriminant metabolites. The distribution of metabolites across treatments (3,5-DCAA, 2,6-DCP-4-CA, and 3,5-DCSA) at (**A**) 12, (**B**) 24, and (**C**) 36 h post-treatment is displayed. While Venn diagrams were generated from all the annotated discriminant metabolites (Table 1), the dendrogram heatmaps were generated from shared metabolites (red arrow) to all three treatments to highlight the differences in the relative concentration at a specific time point. The numerical values in the Venn diagrams correspond to the number of unique metabolites associated with each treatment and the number of overlapping/shared metabolites across the different conditions. Similarly to 12 h, at 36 h, hordatine B was specifically found in extracts from 3,5-DCAA-treated leaves in addition to 12-hydroxyjasmonate sulfate, an isomer of linolenoylglycerol, and other classes of metabolites. Examples of specific metabolites associated with the 3,5-DCSA and 2,6-DCP-4-CA treatments included proline betaine and an OPDA conjugate, respectively. Hordatine B hexose and an isomer of hordatine C were found across all treatments at each time point, and the levels were relatively higher in the 3,5-DCAA samples. Sinapic acid hexose, coumaroylagmatine, 6-prenylnaringenin, 9,12,13-triHODE isomer I, and linolenoyglycerol isomers I and II were all found up-regulated in extracts from 2,6-DCP-4-CA treatments. The relative concentration of sinapoylhydroxyagmatine, saponarin, isovitexin, isovitexin 7,6”-di-O-glucoside, Trp, Leu, and Tyr were down-regulated at 36 h in all treated samples. Here, lutonarin was closely related to 3,5-DCSA and up-regulated in all treated samples. Looking at the dendrogram, similar profiles were observed between DCAA and the control samples and between 2,6-DCP-4-CA and 3,5-DCSA treatments (**C**).

For comparison of all treatments across the chosen time points, Venn diagrams were generated and are represented in Figure 6. At 12 h, among the specific metabolites, coumaroyl- and feruloylagmatine and hordatine B were found as discriminant markers in extracts from 3,5-DCAA-treated leaves, Val, and Ile in 3,5-DCSA-treated samples and isocitric acid in 2,6-DCP-4-CA-treated samples. Hordatines A and B hexose, saponarin, isovitexin 7-O-[X″-feruloyl]-glucoside, and Tyr were all found negatively correlated to every treatment. The dendrogram shows a clear separation between the control and the treated samples (Figure 6A).

At 24 h, metabolites responsible for the uniqueness of each condition involve hordatine C hexose and sinapic acid hexose in extracts from 3,5-DCAA-treated leaves, hordatine C in extracts from 3,5-DCSA-treated leaves and coumaroylputrescine, and sinapoylagmatine in extracts from 2,6-DCP-4-CA-treated leaves. On the dendrogram heatmap, shared metabolites to all three treatments were hydroxytryptamine, coumaroyltryptamine, lutonarin, hordatine B, and its hexosylated form up-regulated in extracts from 3,5-DCAA-treated leaves and closely related to the profile of the 3,5-DCAA treatment. Both coumaroylagmatine and coumaroylhydroxyagmatine were up-regulated in all three conditions but seemed to be more associated with the 2,6-DCP-4-CA treatment. Metabolites closely associated with the 3,5-DCSA treatment were a feruloylagmatine derivative and 6-prenylnaringenin, both down-regulated in the treated samples (Figure 6B).

In Section 2.2, the PCA plots revealed an interesting trend at 24 h, where a clearer sample separation was highlighted between the control and all treatments. Keeping such observation in mind, the relative quantification of barley-important metabolites was evaluated at 24 h. These metabolites were the hordatines and the main flavonoids present in the plant, lutonarin, and saponarin. In addition, two alkaloids, coumaroyltryptamine and hydroxytryptanine, previously not annotated in non-stressed plants [38], were also evaluated (Figure S5). An increase in the biosynthesis of the selected metabolites is observed upon priming, especially with 3,5-DCAA treated samples. Among these, saponarin was by far the most abundant metabolite in the extracts, irrespective of the treatments.

## 4. Discussion

### 4.1. Distribution of Metabolite Classes and Metabolic Pathways Analyses for Biological Interpretation

**Figure 7:**
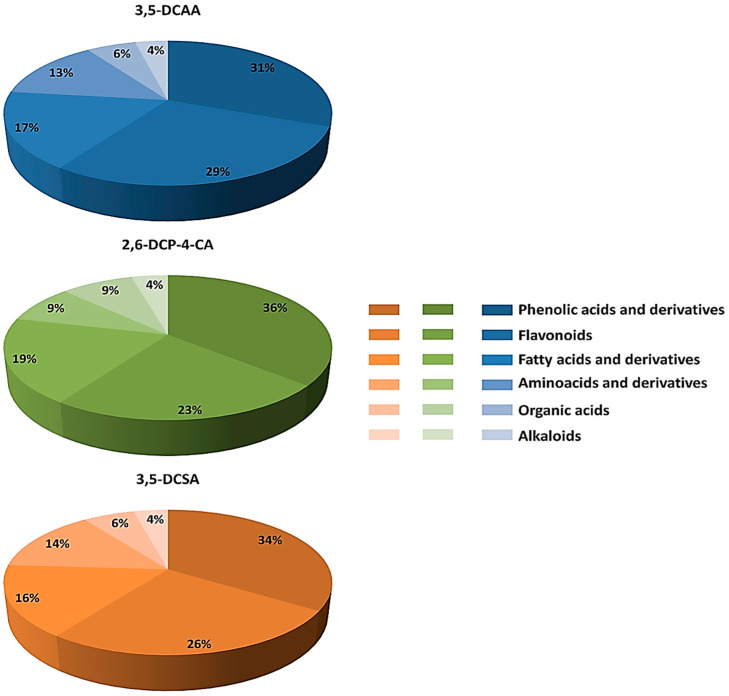
Metabolite class distribution of all annotated discriminant metabolites across all treatments with dichlorinated inducers of acquired resistance. Discriminant metabolites in barley belonged to different classes, as previously identified on the MolNetEnhancer network (Figure 2). Blue: 3,5-DCAA, Green: 2,6-DCP-4-CA, and Orange: 3,5-DCSA.

As discussed above, common and specific responses were observed, featuring fluctuations in metabolites grouped as phenolic acids and derivatives, flavonoids, fatty acids and derivatives, amino acids, organic acids, and alkaloids, listed in descending percentage order across all treatments (Figure 7). These classes belonged to the superfamilies identified on the MolNetEnhancer network (Figure 2). While nuanced differences in the % values were noted between the three treatments, the overall patterns were similar, suggesting that the three dichlorinated inducers (3,5-DCAA, 2,6-DCP-4-CA, and 3,5-DCSA) trigger the same type of response in the barley leaves.

In the environment, barley plants naturally interact with numerous living organisms, instigating a perturbation in the immune system. SA is a key plant immune hormone that is essential for the development of plant immunity. It was one of the first endogenous plant compounds to be documented as an inducer of SAR [31,47,48] and, eventually, metabolic changes. The barley cultivar, ‘Hessekwa’ was treated with synthetic functional analogues of SA, namely, 3,5-DCAA, 2,6-DCP-4-CA, and 3,5-DCSA, and metabolic perturbations and associated reprogramming was evident from PCA models (Figure 3) and in Table 1 and Figure 5 and Figure 6.

**Figure 8:**
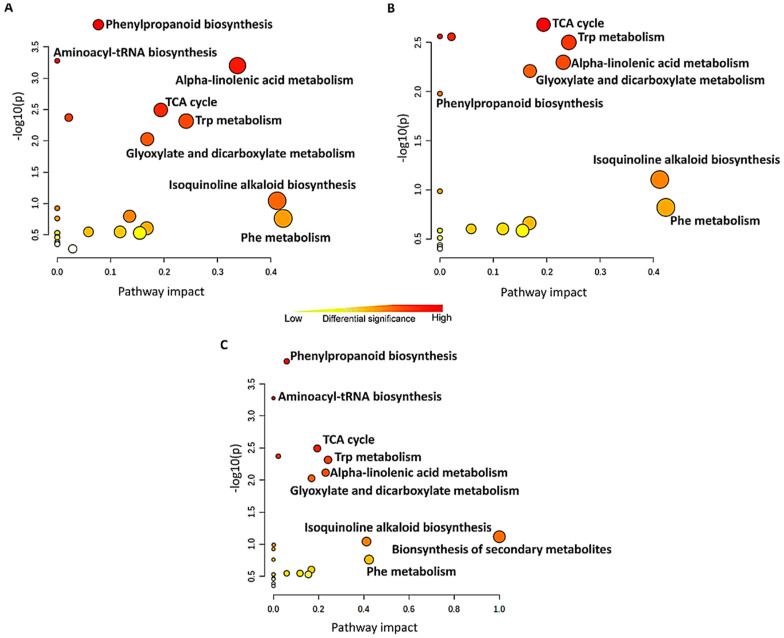
Metabolomic Pathway Analysis (MetPA) as generated by MetaboAnalyst ver. 5.0 with (**A**): 3,5-DCAA, (**B**): 2,6-DCP-4-CA, and (**C**): 3,5-DCSA data. The size of the circle reflects the pathway impact score, while the colour of each circle is based on *p*-values (darker colours imply more significant changes of metabolites in the relevant pathway). TCA: tricarboxylic acid; Trp: tryptophan; Phe: phenylalanine.

Functional analyses (i.e., pathway enrichment analysis) using differential (discriminant) metabolites (Figure 8; Table S1) revealed phenylpropanoid biosynthesis, alpha-linolenic acid metabolism, aminoacyl-tRNA biosynthesis, the TCA cycle, Trp metabolism, Phe, Trp, and Tyr catabolism, and the glyoxylate and dicarboxylate metabolism only to name the most significant and/or the most impactful pathways. These pathways are associated with both primary and secondary metabolism and were identified in all treatments with different levels of significance and impact. These differences can be seen, for example, in the 3,5-DCAA and 3,5-DCSA treatments, where the phenylpropanoid biosynthesis was the most significant pathway and in the case of the 2,6-DCP-4-CA treatment, the TCA cycle. Similar to this study, in tobacco and wheat plants, pathogen- and chemical-induced SAR was characterised by the de novo production of phenylpropanoid pathway chemicals in the leaf, regardless of the signalling pathway [49,50].

### 4.2. Biological Implication with Regard to Plant Protection

In addition to alterations in transcription, the adaptive responses of plants to environmental cues also involve post-translational protein modifications, metabolite alteration, and/or accumulation. These changes all work together to produce a particular physiological response or phenotype. Depending on the system under investigation, chemical and biological priming agents may each exhibit a level of specificity in their action mechanisms that prepares the plant in a different mode [51]. In this context, it was proposed that metabolites that are strongly affected by chemical or biological inducers (the priming agents) are named ‘priming compounds’ since their effect on plant metabolism is to trigger the synthesis of ‘primed compounds’ that assist to counteract the stress. At the same time, these additionally identified metabolites point to defence pathways that the plants deploy to “get ready for the battle” [51].

It has been regarded that a primary metabolism only performs a supportive role in plant defence and that the energy saved by the down-regulation of primary metabolism is diverted and used for defence responses. However, the up-regulation of metabolic pathways associated with primary metabolism may also occur during plant defence, and it was proposed that such up-regulation modulates signal transduction cascades that lead to plant defence responses [52]. Amino acids participate in plant growth and development, signalling processes, and stress responses. In addition to being building blocks for protein biosynthesis, they also perform pivotal roles in several pathways associated with secondary metabolism [53,54]. Free amino acids are essential for SAR in plants, and their presence has previously been attributed to the plant’s tolerance [55,56]. The negative correlation between the Tyr, Phe, and Trp levels and the treatments observed in this study might be attributed to the rapid use of the compounds in the biosynthesis of secondary metabolites involved in plant defence, and for de novo protein synthesis as in the case of pathogenesis-related (PR) proteins. For instance, Phe is the substrate of the first committed step in the phenylpropanoid pathway (the most significant pathway to respond to 3,5-DCAA and 3,5-DCSA, Figure 8), which results in the biosynthesis of phenolic compounds, including hydroxycinnamic acids (HCAs) and flavonoids. In addition, amino acids can be converted into precursors or intermediates in the TCA cycle, supporting the mitochondrial metabolism and the production of ATP [57,58]. Consequently, it can be speculated that there is an imbalance in the ratio between amino acid anabolism and catabolism. The level of amino acids produced might not be proportional to the plant’s requirement for energy and other processes.

Trp in barley seedlings is a precursor to simple indole alkaloids, gramine, and tryptamine. Coumaroyltryptamine and hydroxytryptamine were found to be discriminatory and positively correlated to the treatments (Figure S5; Table 1). The decrease in Trp could also be linked to the accumulation of tryptamine derivatives which participate in plant protection. In previous studies, tryptamine and derivatives accumulated in the member of the grass family (Poaceae species) following the application of compounds, such JA as and fungal pathogens [59,60]. The reliance of both the constitutive and inducible defence mechanisms on the Trp pathway was previously demonstrated [60].

Commenting on energy, functional analogues of SA are used to activate induced resistance; however, this process consumes a lot of energy and interferes with other metabolic functions. Therefore, balancing the trade-off between defence and growth may be key to a plant’s success [20]. Alterations in the production of organic acids, crucial contributors to energy production, were noted, and the TCA cycle was one of the most significant pathways observed, especially in the 2,6-DCP-4-CA treatment. There was a positive correlation between malic acid with 2,6-DCP-4-CA and 3,5-DCSA treatments at 36 and 12 h, respectively. Citric and isocitric acid were consistently found as discriminative metabolites and negatively correlated to all treatments at 24 h. In Pastor et al., 2014, [51], the TCA cycle was activated and potentiated in β-aminobutyric acid (BABA)-primed Arabidopsis plants. Organic acids are essential to all plant species as they constitute good storage for carbon, perform a role in CO_2_ fixation and stomatal conductance, help plants deal with excess cations, and are reversibly implicated in the biosynthesis of amino acids and other compounds [61,62]. Specifically, citrate is produced in the TCA cycle from the condensation of oxaloacetate, the end product of a previous turn of the cycle, and acetyl-CoA and provides a bridge between carbohydrate and fatty acid metabolism.

The majority of fatty acid derivatives were positively correlated to the treatments at 12 and 36 h. These compounds are alternative sources of energy for plant growth and development. Of special interest and part of discriminant metabolites are the JA derivatives, such as 12-hydroxy-JA sulphate, only found in the 3,5-DCAA treatment, and dihydro-JA found in both 2,6-DCP-4-CA and 3,5-DCSA treatments. Moreover, a conjugate of OPDA, the precursor of JA, was also increased due to the 2,6-DCPCA and 3,5-DCSA treatments. Jasmonates are by-products of the classical octadecanoid oxylipin pathway deriving from alpha-linolenic acid metabolism, identified here as a significant metabolic pathway [63,64]. Recent studies have elaborated on the direct and active roles of fatty acids and their breakdown products on various defence mechanisms [65]. They are involved in the regulation of plants’ basal, effector-triggered, and systemic immunity, and perform an important role in NADPH oxidase activation which result in the biosynthesis of reactive oxygen species (ROS). In addition to regulating plant growth and development, JA is crucial in the resistance against both biotic and abiotic stresses. It acts as a mobile signal for SAR and is translocated via vasculature [66]. Despite being clearly characterised by the molecules they synthesise, JA, SA, and ET signalling pathways interact both cooperatively and antagonistically in a range of responses [13,66]. While the first reports elaborated on the inhibitory effect of SA on JA actions in tomatoes, positive crosstalk between the two phytohormones has also been highlighted in a number of systems [67,68]. These hormones were not annotated in the study; however, as mentioned above, precursors of JA and JA derivatives were annotated, suggesting the activation of this pathway and a state of alertness (primed state).

Phenolic acids and conjugated derivatives are end products in the phenylpropanoid pathway. The activation of the phenylpropanoid pathway is associated with enhanced resistance to stresses. The chlorogenic acid, 4-O-*p*-coumaroylquinic acid, was found up-regulated in 3,5-DCAA treated plants at 36 h. Sinapic acid hexose accumulated in all treated plants at 12 and 36 h. Pastor et al. [51] hypothesised that phenolics and sinapates are compounds produced in primed plants that support the biosynthesis of metabolites functioning in cell wall reinforcement. Coumaroylagmatine, a well-known precursor of hordatine A and B, was positively correlated to all treatments at 24 and 36 h. Feruloyagmatine and sinapolyagmatine, on the other hand, were only found to be positively correlated to 2,6-DCP-4-CA at 24 h and 3,5-DCAA at 36 h, respectively. Increased production of HCAAs was reported in maize after insect herbivory attack [69,70]. In a study by [58], the HCAA coumaroylputrescine accumulated in barley leaves treated with JA. Here, the positive correlation of the compound to the 2,6-DCP-4-CA treatment at 24 h was also observed. Similarly, to the precursors, hordatine A, B, and C production, was increased mostly at 24 h in both 3,5-DCAA and 3,5-DCSA (Figure S5; Table 1, and hordatine B at 36 h in the case of the 2,6-DCP-4-CA treatment (Table 1). The synchronised increase in the relative content of both hordatines and precursors highlights the role of both the phenylpropanoid- and polyamine pathways in plant immunity. The role of these barley-specific metabolites in antimicrobial defences was previously highlighted [37,38], and the increase in their production can be related to the preconditioning of the plants. From the time point at which the increase in the production of hordatines and precursors occurred, it might be speculated that in the ‘Hessekwa’ cultivar it takes at least 24 h to induce a positive correlation to the treatment. In terms of specific markers for each treatment in this class of metabolites, a derivative of *p*-coumaric acid, 4-O-*p*-coumaroylquinic acid, and *p*-coumaroylputrescine were only found up-regulated by 3,5-DCSA, 3,5-DCAA, and 2,6-DCP-4-CA, respectively. It might be suggested that each treatment uses different molecules to perform similar functions.

Flavonoids are structurally related compounds performing crucial roles in biotic and abiotic stresses [71], and in transcriptional and growth regulation [72]. The glycosylated derivatives of luteolin and apigenin (lutonarin and saponarin, respectively) are the most dominant flavonoids in barley and were annotated as discriminant metabolites in all treatments at almost all time points. While lutonarin was always up-regulated, saponarin levels increased only at 12 and 24 h in samples from 3,5-DCSA treated plants and at 24 h with 3,5-DCAA treatment. Both compounds are good antioxidants [73] and are proposed as protectants against UV-B radiation [74]. The aglycones have very similar chemical structures and do not differ meaningfully in their antibacterial activity. Likewise, the presence and location of the sugar group(s) in the flavone glucosides do not have a significant effect on the antibacterial activity [75]. Isovitexin 2′-O-glucose was up-regulated only at 24 h and down-regulated at 12 and 36 h, while 6-prenylnaringenin followed the opposite pattern. Here, the diurnal and nocturnal responses might influence the pattern of accumulation since some mechanisms are activated in the presence of light and others in the absence.

Several studies have demonstrated that induction of plant immune and defence responses can be associated with enhanced expression of the phenylalanine ammonia-lyase (*PAL*) gene(s). PAL is the first enzyme in the phenylpropanoid pathway and is responsible for the production of phenolic compounds, such as phenolic acids and derivatives, and flavonoids. The fluctuation of these compounds in the plants is evidence of the plants’ reaction to the treatments. Despite the fact that SA is the only specific phenolic molecule that is extensively used as a biochemical marker for SAR, the implication of phenolics in the identification of plant damage and plant signalling during the development of SAR is acknowledged. In wheat, DCPCA significantly increased the phenolic content [42]. In fact, the first artificial substances that were demonstrated to activate SAR were 2,6-DCINA acid and its methyl ester [19,76,77]. In tobacco, 2,6-DCINA and 3,5-DCSA were reported to be efficient at enhancing PR1 protein expression and resistance against the tobacco mosaic virus (TMV) [34,77]. Compared to SA, the dichlorinated 3,5-DCSA was found to be more active at inducing resistance against TMV [32]. 3,5-DCAA has been established as an inducer of transient and rapid resistance to pathogens in Arabidopsis by simultaneously engaging two different branches of the plant defence signalling network: NPR1-dependent and NPR1-independent responses [28]. The interaction of 3,5-DCAA with defence signalling pathways takes place either downstream or independently of SA perception and accumulation. Here, in the absence of SA-related metabolites as discriminant biomarkers, it might be speculated that these treatments in barley, or at least in the ‘Hessekwa’ cultivar, are not SA-dependent. In priming, multiple layers of induced defence mechanisms can be involved. Enhanced responses developed upon treatment with these inducers were mostly observable through the activation of the TCA cycle, phenylpropanoid pathway, and alpha-linolenic acid metabolism, converting the metabolome of the naïve plants to a primed state. This was attained by the fluctuation of common and specific protective metabolites, such as the accumulation of JA conjugates. Due to the nature of untargeted metabolomics, it is difficult to distinguish between ‘priming agents’ and ‘primed compounds’ [51], but the annotated metabolites and the identified metabolic pathways were supportive of a defensive role and its link to priming. The investigation of molecular and biochemical mechanisms in priming events is still ongoing [78], and this study is a contribution in that regard.

## 5. Conclusions

Timeous activation of defence mechanisms has proven to be of great importance for the plant’s survival under environmental stressors. In this context, the fluctuation of several metabolites was observed on the shoot tissue of the barley cultivar ‘Hessekwa’ using xenobiotic dichlorinated substitutes of salicylic acid, anthranilic acid, and isonicotinic acid (3,5-DCSA, 3,5-DCAA, and 2,6-DCP-4-CA/2,6-DCINA). It is known that even a subtle change in the substitution site of a compound can significantly affect the physicochemical properties thereof and, ultimately, the biological activity; this was reiterated in this study. 3,5-DCAA, 2,6-DCP-4-CA, and 3,5-DCSA were proposed as chemicals with either induced/acquired resistance or priming activity. There is a relationship between the two concepts, and while there is considerable overlap between the phenotypes of the SAR and ISR states, the underlying triggers and mechanisms of action differ. With priming inducers, it has recently become apparent that defence priming should be regarded as an adaptive part of induced resistance and that specific defence mechanisms also depend strongly on the primed state. The question has been raised whether metabolism (and thus also metabolites, i.e., the ‘primed compounds’) can store and process information regarding induced/imprinted/primed responses to changing environments.

Applying untargeted metabolomics approaches, specific and, interestingly, shared mechanisms were highlighted; notably the activation of the phenylpropanoid pathway, the TCA cycle, and the alpha-linolenic acid metabolism by all three inducers. With regard to metabolites annotated in the study, it is important to note that in some cases, they were either conjugated compounds, substrates, or precursors in the biosynthesis of the main components in plant immunity. For example, no signalling molecules (SA, JA, and ET) were present as discriminant metabolites. Instead, JA derivatives and oxylipins, key components in the phytohormone biosynthesis, were found to be discriminative and were mostly up-regulated in treated samples conditions. The presence of related compounds but not the main actors themselves can be perceived as the plant’s readiness to quickly produce more specific defence-related metabolites in response to environmental stress (e.g., pathogen attacks). It was then suggested that plants use metabolic imprints (i.e., the metabolic changes that last beyond recovery from stress events) and priming (e.g., the imprints that function to prepare for upcoming stresses) to integrate diverse environmental stress histories. The lack of SA accumulation in this study led to the speculation that the mechanism of priming by dichlorinated xenobiotics (at least in the ‘Hessekwa’ cultivar of barley) does not rely on SA and derivatives but rather on JA and derivatives. Hordatines (known phytoanticipins/phytoalexins in barley), their hexosylated conjugates and precursor molecules were also found as important discriminant markers of inducer treatment with a time-lapse of 24 h required to enhance the biosynthesis. In addition, the flavonoid profile was also altered and specific markers were apigenin 6-C-arabinoside 8-C-glucoside, isoscoparin 7-O-[6″-sinapoyl]-glucoside, and isovitexin 2″-O-glucoside, associated with the 3,5-DCAA-, 2,6-DCP-4-CA-, and 3,5-DCSA-treatments, respectively. Although examples of flavanones and flavonols were amongst the discriminant metabolites, the large number of flavones (as glycosidic derivatives of apigenin and luteolin) were especially prevalent. The increase in the production of tryptamine derivatives, precursors in the biosynthesis of various indole alkaloids, pointed to their participation in defence-related mechanisms and their contribution to the preconditioned state.

Metabolomics approaches, combined with chemometric analysis, have proven to be valuable tools in revealing the metabolic perturbation and reprogramming resulting from the above-mentioned treatments. Chemometrics tools allowed the exploration of large datasets and also to discriminate among the different conditions. All three dichlorinated xenobiotic priming inducers were good candidates in perturbing plant metabolic pathways associated with metabolites involved in defence. Although presenting specific markers, the mechanisms of action seem to be similar. Regardless of nuanced differences, their effects appear to work toward the same goal of establishing an enhanced defensive environment.

## Figures and Tables

**Table 1:** List of all annotated (putatively identified) metabolites extracted from leaves of the barley cultivar ‘Hessekwa’ treated with 3,5-DCAA, 2,6-DCP-4-CA, and 3,5-DCSA, and harvested at 12, 24, and 36 h post-treatment. The compounds were extracted from the OPLS-DA S-plots, and the fold changes were calculated using a SIMCA software algorithm and are indicated where a metabolite was selected as a significant biomarker. Blue: 3,5-DCAA, Green: 2,6-DCP-4-CA, Orange: 3,5-DCSA, Purple: Control. Different shades of the same colour indicated a specific time point (ranging from lighter to darker, with the former corresponding to 12 h and the latter to 36 h; e.g., 3,5-DCAA 12 h was compared to Control 12 h and metabolites with a FC > 1 were shaded with light blue while those with a FC < 1 were shaded with purple.

	ESI Mode	Compounds	Rt (min)	*m*/*z*	DCAA Fold Change			DCPCA Fold Change			DCSA Fold Change		
					12 h	24 h	36 h	12 h	24 h	36 h	12 h	24 h	36 h
1	–	*p*-Coumaric acid derivative	0.86	404.103	●	●	●	●	●	●	1.48	1.18	1.16
2	–	4-O-*p*-Coumaroylquinic acid	1.15	337.084	●	●	5.58	●	●	●	●	●	●
3	–	3-Hydroxycoumarin	1.25	161.043	●	●	●	●	0.58	●	●	●	●
4	–	3-O-*p*-Coumaroylquinic acid	3.06	337.112	●	●	●	●	0.83	●	●	0.78	
5	–	Sinapic acid hexose	5.14	385.113	●	0.65	2.18	2.21	●	4.59	1.86	●	2.12
6	–	Dihydroferulic acid 4-O-glucuronide	7.01	371.096	0.87	0.84	1.087	●	●	●	0.82	0.84	●
7	+	Coumaroylputrescine	2.39	235.145	●	●	●	●	1.68	●	●	●	●
8	+	Coumaroylhydroxyagmatine	2.57	293.157	●	1.37	1.27	●	1.45	●	●	1.15	1.09
9	+	Coumaroylagmatine	4.06	277.161	0.35	1.33	1.09	●	1.62	1.17	●	1.34	1.07
10	–	Feruloylhydroxyagmatine	4.49	323.133	●	0.73	●	●	0.84	●	●	0.74	●
11	+	Feruloylagmatine	5.30	307.172	0.612	●	●	●	●	1.14	●	●	●
12	+	Sinapoylagmatine	6.17	337.186	●	●	3.08	●	0.32	●	●	●	●
13	–	Sinapoylhydroxyagmatine	6.30	351.126	●	0.93	0.93	0.88	●	0.89	●	0.92	0.92
14	–	Hordatine B hexose	3.81	787.364	0.818	1.14	1.17	0.74	1.10	0.91	0.78	1.16	1.22
15	–	Hordatine A hexose	4.14	757.353	0.80	●	●	0.63	1.12	0.80	0.75	●	●
16	–	Hordatine C hexose isomer I	4.63	817.376	●	●	●	0.68	1.24	●	0.72	1.31	●
17	–	Hordatine C hexose isomer II	6.26	771.200	●	1.92	●	●	●	●	●	●	●
18	–	Hordatine B isomer I	7.28	579.304	0.63	1.13	1.11	0.86	0.67	●		1.14	●
19	+	Hordatine A Isomer I	7.72	551.304	0.01	1.34	●	0.85	●	●	●	1.32	0.92
20	–	Hordatine A isomer II	7.76	549.294	●	●	●	●	●	2.61	●	●	3.49
21	+	Hordatine B isomer II	7.97	581.319	0.06	1.41	0.82	●	●	●	●	1.50	●
22	+	Hordatine C + 46 isomer I	7.99	655.320	0.83	1.09	1.12	0.87	●	0.88	●	1.17	0.92
23	+	Hordatine C isomer II	8.67	611.330	●	●	●	●	●	●	●	1.38	●
24	–	Isoorientin 7-O-glucoside(Lutonarin)	6.43	609.144	1.45	1.81	1.11	●	1.22	1.13	1.35	1.24	1.15
25	–	Isoorientin 7-O-[6″-sinapoyl]-glucoside	10.53	815.205	1.40	1.26	●	●	0.73	●	●	●	●
26	–	Isovitexin 7,6″-di-*O*-glucoside	8.15	755.205	●	●	0.85	0.53	●	0.55	●	●	0.73
27	+	Isovitexin -7-O-glucoside (Saponarin)	8.39	595.166	0.56	1.21	0.78	0.93	●	0.87	1.06	1.31	0.87
28	–	Isovitexin 7-O-rhamnosylglucoside	8.81	739.208	1.09	0.93	0.89	0.91	●	0.86	●	●	●
29	–	Isovitexin 2″-O-glucoside	9.79	593.150	●	●	●	●	●	●	●	0.01	10.97
30	–	Isovitexin 2″-O-arabinoside	9.93	563.139	●	0.01	6.34	●	●	●	●	0.01	11.45
31	–	Isovitexin	10.59	431.097	0.66	●	0.29	0.40	2.09	0.13	●	2.02	0.43
32	–	Isovitexin 7-O-[6″-sinapoyl]-glucoside	11.42	799.210	1.16	0.77	1.18	1.09	0.65	1.47	●	0.70	●
33	–	Isovitexin 7-O-[X″-feruloyl]-glucoside (Feruloylsaponarin)	11.82	769.200	0.89	0.93	●	0.85	●	●	0.84	●	●
34	–	Apigenin 7-O-arabinosylglucoside	11.90	563.140	●	●	0.82	●	●	●	●	●	0.85
35	–	Apigenin 6-C-arabinoside 8-C-glucoside	8.97	563.140	1.52	●	●	●	●	●	●	●	●
36	–	Isoscoparin 7-O-glucoside	8.99	623.160	●	1.31	0.85	●	1.29	0.78	1.10	1.26	●
37	+	Isoscoparin 2″,6”-di-*O*-glucoside	10.89	787.209	●	1.53	●	●	1.29	●	●	●	●
38	–	Isoscoparin 7-O-[6″-sinapoyl]-glucoside	11.53	829.221	●	●	●	0.72	●	●	●	●	●
39	–	Isoscoparin 7-O-[6″-feruloyl]-glucoside	11.95	799.211	●	●	●	●	1.32	0.72	●	1.25	●
40	–	Isorhamnetin-3-O-glucoside	9.74	477.107	●	3.49	●	●	●	●	●	2.43	●
41	–	6-Prenylnaringenin	19.02	339.215	●	0.19	0.68	5.42	0.82	3.60	3.07	0.53	1.80
42	+	Hydroxytryptamine	1.67	177.102	●	2.47	2.50	●	1.31	●	1.84	1.76	1.26
43	–	Coumaroyltryptamine	2.60	289.129	●	1.29	1.55	0.76	1.19	●	1.15	1.14	1.15
44	+	Valine	0.88	118.086	●	●	0.77	●	●	●	1.26	●	●
45	+	Tyrosine	1.14	182.081	0.50	●	0.72	0.70	●	0.58	0.63	●	0.61
46	+	Tyrosine derivatives	1.14	276.107	0.33	1.60	●	●	●	●	●	●	●
47	+	Isoleucine	1.31	132.102	●	●	0.74	●	0.72	0.55	0.61	●	0.69
48	+	Phenylalanine	1.65	166.087	●	0.82	0.58	0.64	0.743	0.44	0.51	0.76	0.52
49	+	Proline betaine	2.05	144.139	●	●	●	●	●	●	●	●	15.26
50	–	Tryptophan	2.43	203.080	●	●	0.84	0.76	0.65	0.65	0.71	0.74	0.63
51	–	Asparaginylglucose	4.24	293.122	●	0.79	●	●	●	●	●	0.81	●
52	–	Citric acid	1.14	191.017	●	0.55	●	●	0.50	●	●	0.49	●
53	–	Isocitric acid	0.93	191.017	●	0.69	●	1.29	0.42	●		0.41	●
54	–	Citric acid derivative	1.39	306.117	●	●	●	●	0.85	●	●	●	●
55	–	Malic acid	0.95	133.012	●	●	0.74	●	0.74	1.22	1.27	0.73	●
56	–	12-Hydroxyjasmonate sulfate	16.61	305.129	●	●	2.93	●	●	●	●	●	●
57	–	Dihydrojasmonic acid (H_2_JA) conjugate	16.80	419.173	●	●	●	●	●	2.14	●	●	1.34
58	–	9,12,13,TriHODE isomer I	16.53	327.216	●	0.39	0.70	3.52	●	2.23	1.67	0.61	1.69
59	–	9,12,13,TriHODE Isomer II	16.64	327.216	●	●	●	10.73	●	●	●	●	●
60	–	TriHOME	17.25	329.232	●	0.31	●	4.59	●	●	2.13	0.46	●
61	–	9-Oxo-12,13-diHODE	17.45	325.200	●	0.14	●	6.31	●	3.51	2.41	0.43	2.27
62	–	OPDA conjugate	19.55	309.205	●	0.23	●	3.90	●	2.47	3.73	0.31	●
63	+	Linolenoylglycerol isomer I	20.69	353.267	●	●	2.12	1.93	●	3.08	1.71	●	1.71
64	+	Linolenoylglycerol isomer II	20.98	353.265	●	●	1.70	●	●	2.71	1.64	●	1.70
65	+	Linolenoylglycerol isomer III	21.80	353.263	●	●	1.72	●	●	●	●	●	●
66	+	Linolenoylglycerol isomer VI	22.07	353.263	2.57	●	●	●	●	1.63	2.17	0.70	●

• = annotated in MS chromatograms, (+) ESI positive mode, (–) ESI negative mode.

## Data Availability

The study design information, LC-MS data, data processing and analyses are reported and incorporated into the main text. Raw data, analyses, data processing information, and the meta-data were deposited to the EMBL-EBI metabolomics repository—MetaboLights50, on 12 January 2023 with the identifier MTBLS6874 (www.ebi.ac.uk/metabolights/MTBLS6874).

## Supplementary Materials

The following are available online at https://www.mdpi.com/article/10.3390/metabo13050666/s1, Figure S1. Ultra–high performance liquid chromatography–mass spectrometry (UHPLC–MS) base peak intensity (BPI) chromatograms (negative ionisation) of shoot extracts from the ‘Hessekwa’ cultivar treated with DCAA, DCPCA, and DCSA for 12, 24, and 36 h. (Comparison of time-dependent changes). Figure S2. Ultra–high performance liquid chromatography–mass spectrometry (UHPLC–MS) base peak intensity (BPI) chromatograms (positive ionisation) of shoot extracts from the ‘Hessekwa’ cultivar treated with DCAA, DCPCA, and DCSA for 12, 24, and 36 h. (Comparison of time-dependent changes). Figure S3. Ultra–high performance liquid chromatography—mass– spectrometry (UHPLC–MS) base peak intensity (BPI) chromatograms (negative ionisation) of shoot extracts from the ‘Hessekwa’ cultivar treated with DCAA, DCPCA, and DCSA and harvested after 12 h, 24 h, and 36 h. (Comparison according to inducer). Figure S4. Principal component analysis (PCA) score plot models of ESI (+) data from shoot extracts of the ’Hessekwa’ cultivar of *Hordeum vulgare*. Figure S5. Relative quantification (based on average peak area values) of selected primed/induced metabolites at 24 h. Hordatine A, B, and C; flavones lutonarin, saponarin, and alkaloids hydroxytryptamine and coumaroyltryptamine. Table S1. Metabolic pathways generated from Metabolomics Pathway Analysis (MetPA) in MetaboAnalyst 5.0 involve selected annotated metabolites. Blue: 3,5-DCAA; Green: 2,6-DCP-4-CA, and Orange: 3,5-DCSA.
