# Supplementary material for: Metabolic Reprogramming of Barley in Response to Foliar Application of Dichlorinated Functional Analogues of Salicylic Acid as Priming Agents and Inducers of Plant Defence
Source: Metabolites. 2023 May 17;13(5):666. doi: 10.3390/metabo13050666 (PMC10223260; doi:10.3390/metabo13050666)
Supplement: Supplementary file 1 [file metabolites-13-00666-s001.zip › metabolites-2389588-supplementary.pdf]

Metabolites

Supplementary File

Metabolic Reprogramming of Barley in Response to Foliar Application of Dichlorinated Functional Analogues of Salicylic Acid as Priming Agents and Inducers of Plant Defence

Claude Y. Hamany Djande, Paul A. Steenkamp, Lizelle A. Piater, Fidele Tugizimana and Ian A. Dubery *

Research Centre for Plant Metabolomics, Department of Biochemistry, University of Johannesburg, P.O. Box 524, Auckland Park, Johannesburg 2006, South Africa; claudeh@uj.ac.za (C.Y.H.D.); psteenkamp@uj.ac.za (P.A.S.); lpiater@uj.ac.za (L.A.P.); ftugizimana@uj.ac.za (F.T.)

* Correspondence: idubery@uj.ac.za; Tel.: +27-11-5592401

Figure S1. Ultra–high performance liquid chromatography - mass spectrometry (UHPLC–MS) base peak intensity (BPI) chromatograms (negative ionisation) of shoot extracts from the 'Hessekwa' cultivar treated with DCAA, DCPCA and DCSA for 12, 24 and 36 h. (Comparison of time-dependent changes).

Figure S2. Ultra–high performance liquid chromatography - mass spectrometry (UHPLC–MS) base peak intensity (BPI) chromatograms (positive ionisation) of shoot extracts from the 'Hessekwa' cultivar treated with DCAA, DCPCA and DCSA for 12, 24 and 36 h. (Comparison of time-dependent changes).

Figure S3. Ultra–high performance liquid chromatography - mass spectrometry (UHPLC–MS) base peak intensity (BPI) chromatograms (negative ionisation) of shoot extracts from the 'Hessekwa' cultivar treated with DCAA, DCPCA and DCSA and harvested after 12 h, 24 h and 36 h. (Comparison according to inducer).

Figure S4. Principal component analysis (PCA) score plot models of ESI (+) data from shoot extracts of the 'Hessekwa' cultivar of *Hordeum vulgare*.

Table S1. Metabolic pathways generated from Metabolomics Pathway Analysis (MetPA) in MetaboAnalyst 5.0 and involving selected annotated metabolites. Blue: 3,5-DCAA; Green: 2,6-DCP-4-CA and Orange: 3,5-DCSA.

Figure S5. Relative quantification (based on average peak area values) of selected primed / induced metabolites at 24 h. Hordatine A, B and C; flavones lutonarin, saponarin and alkaloids hydroxytryptamine and coumaroyltryptamine.

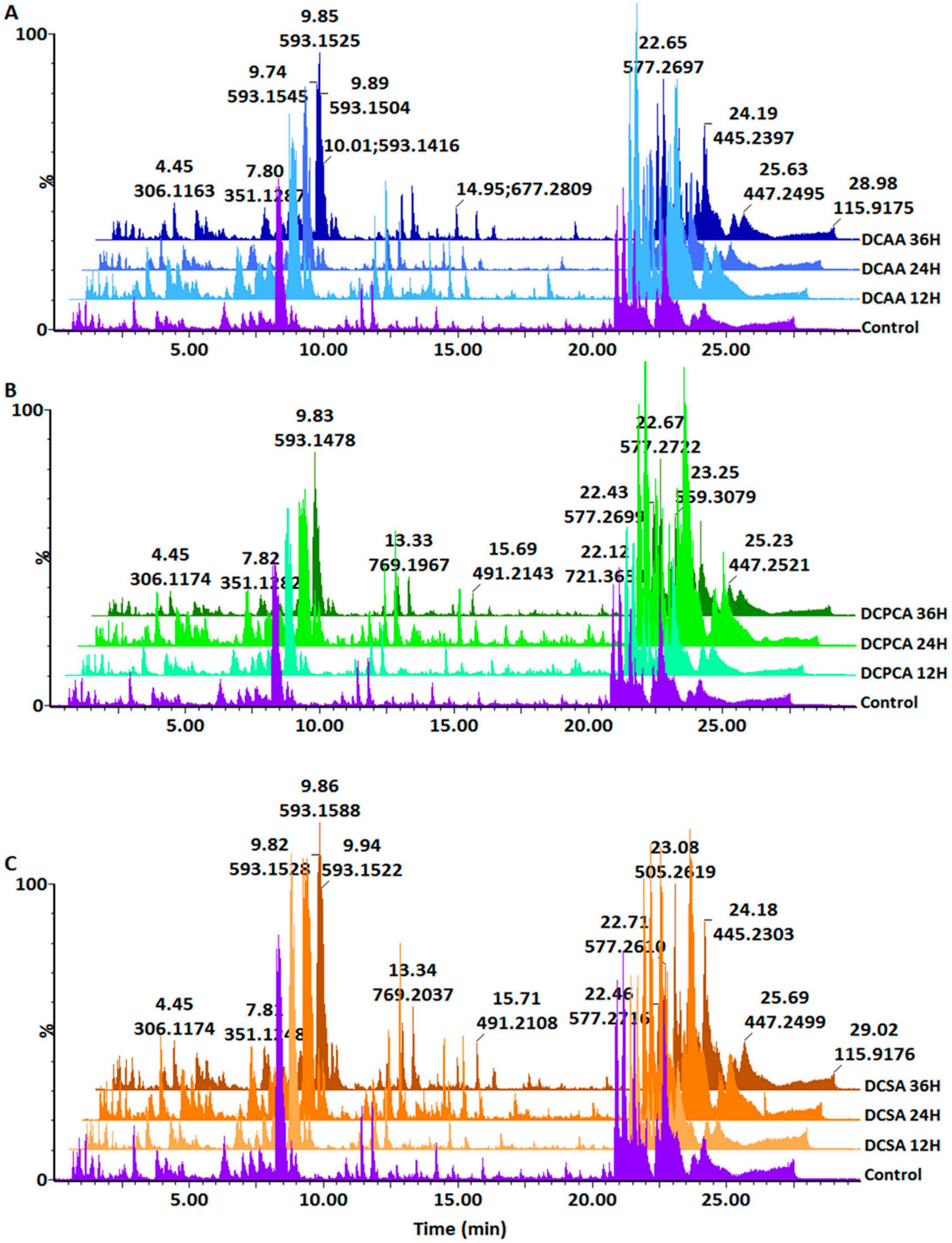

Figure S1. Ultra–high performance liquid chromatography - mass spectrometry (UHPLC–MS) base peak intensity (BPI) chromatograms (negative ionisation) of shoot extracts from the 'Hessekwa' cultivar treated with DCAA (**A**), DCPCA (**B**) and DCSA (**C**) for 12, 24 and 36 h. (Comparison of time-dependent changes).

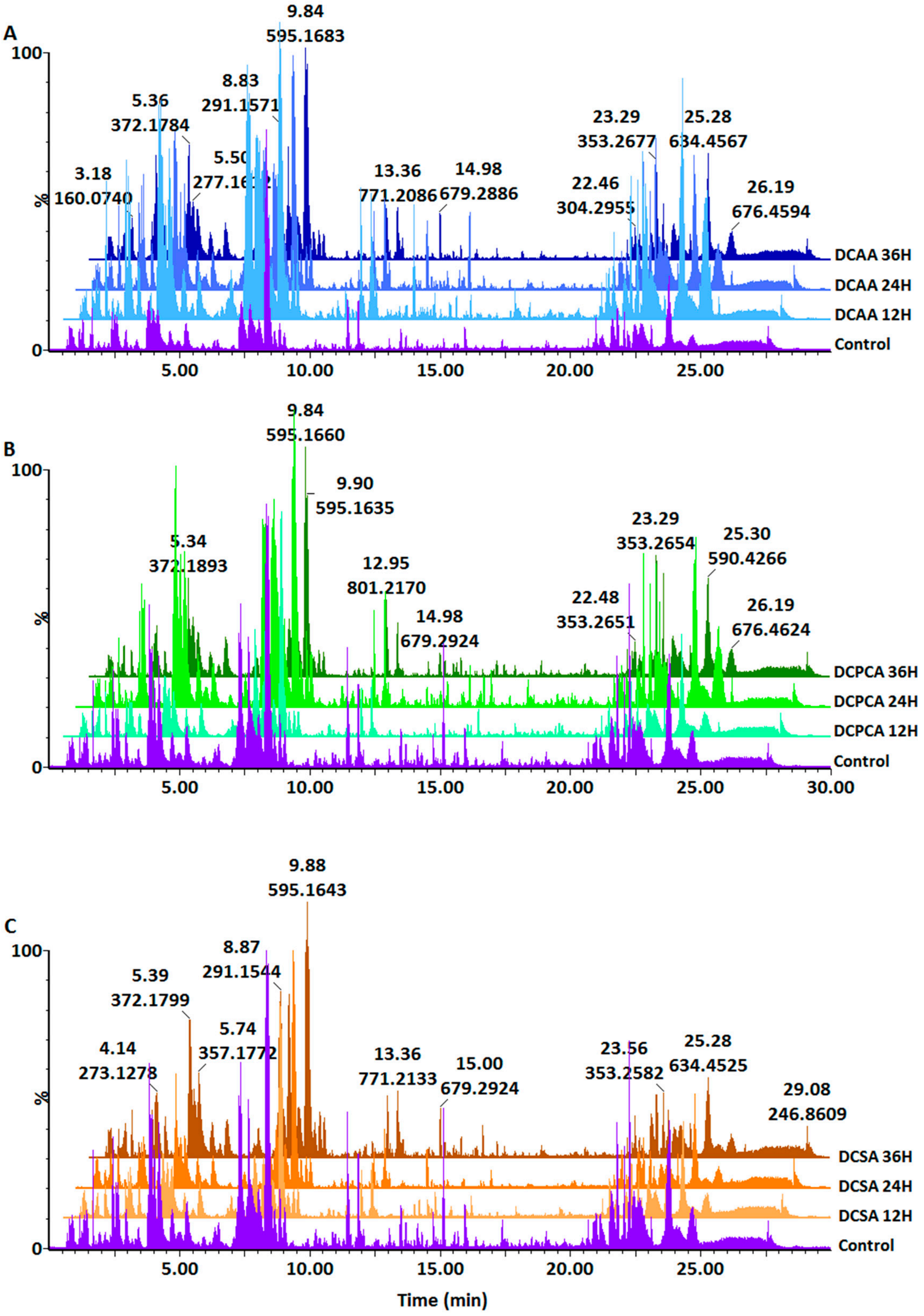

Figure S2. Ultra–high performance liquid chromatography - mass spectrometry (UHPLC–MS) base peak intensity (BPI) chromatograms (positive ionisation) of shoot extracts from the 'Hessekwa' cultivar treated with DCAA **(A)**, DCPCA **(B)** and DCSA **(C)** for 12, 24 and 36 h. (Comparison of time-dependent changes).

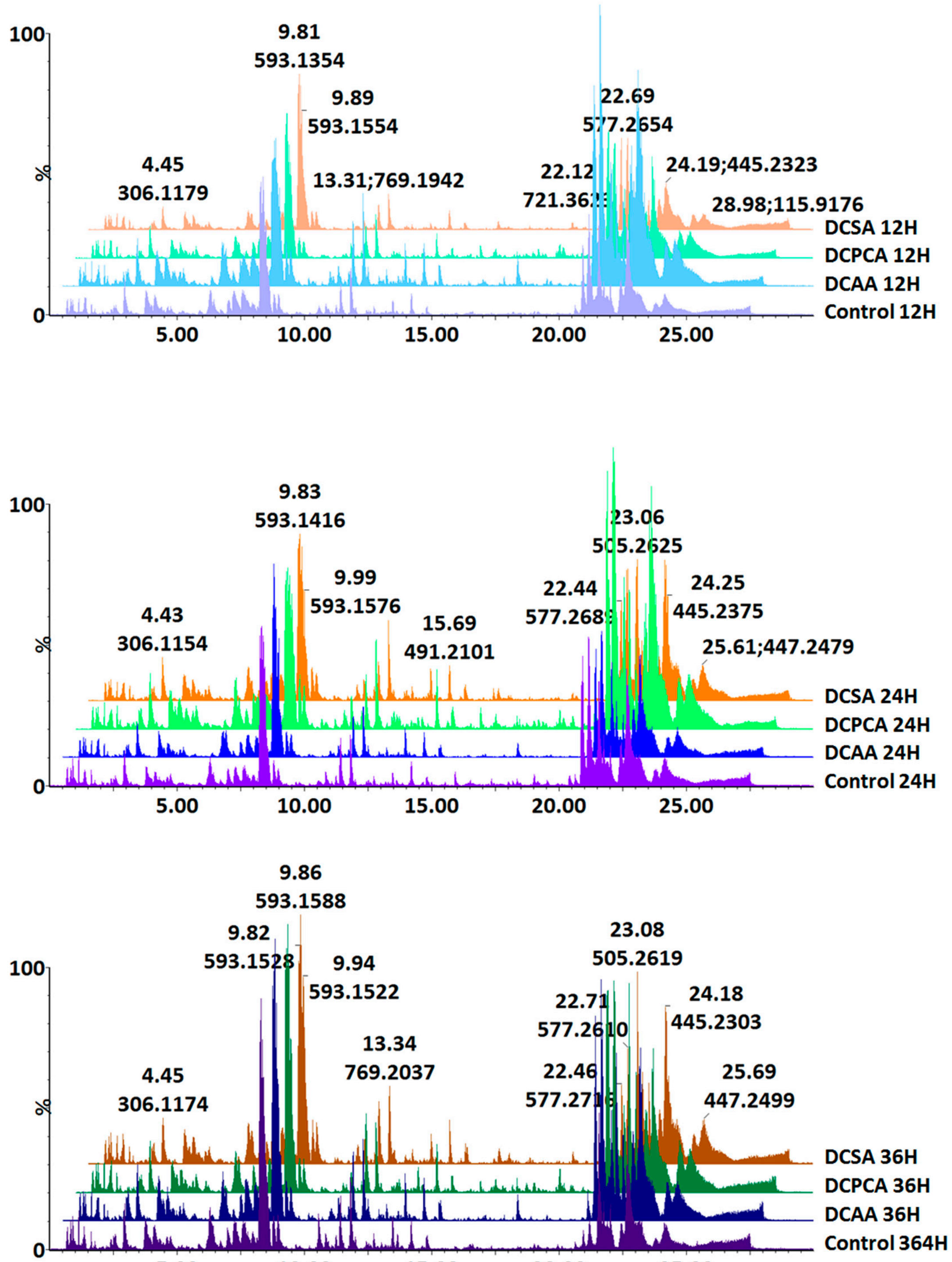

Figure S3. Ultra–high performance liquid chromatography - mass spectrometry (UHPLC–MS) base peak intensity (BPI) chromatograms (negative ionisation) of shoot extracts from the 'Hessekwa' cultivar treated with DCAA, DCPCA and DCSA and harvested after 12 h (**A**), 24 h (**B**) and 36h (**C**). (Comparison according to inducer).

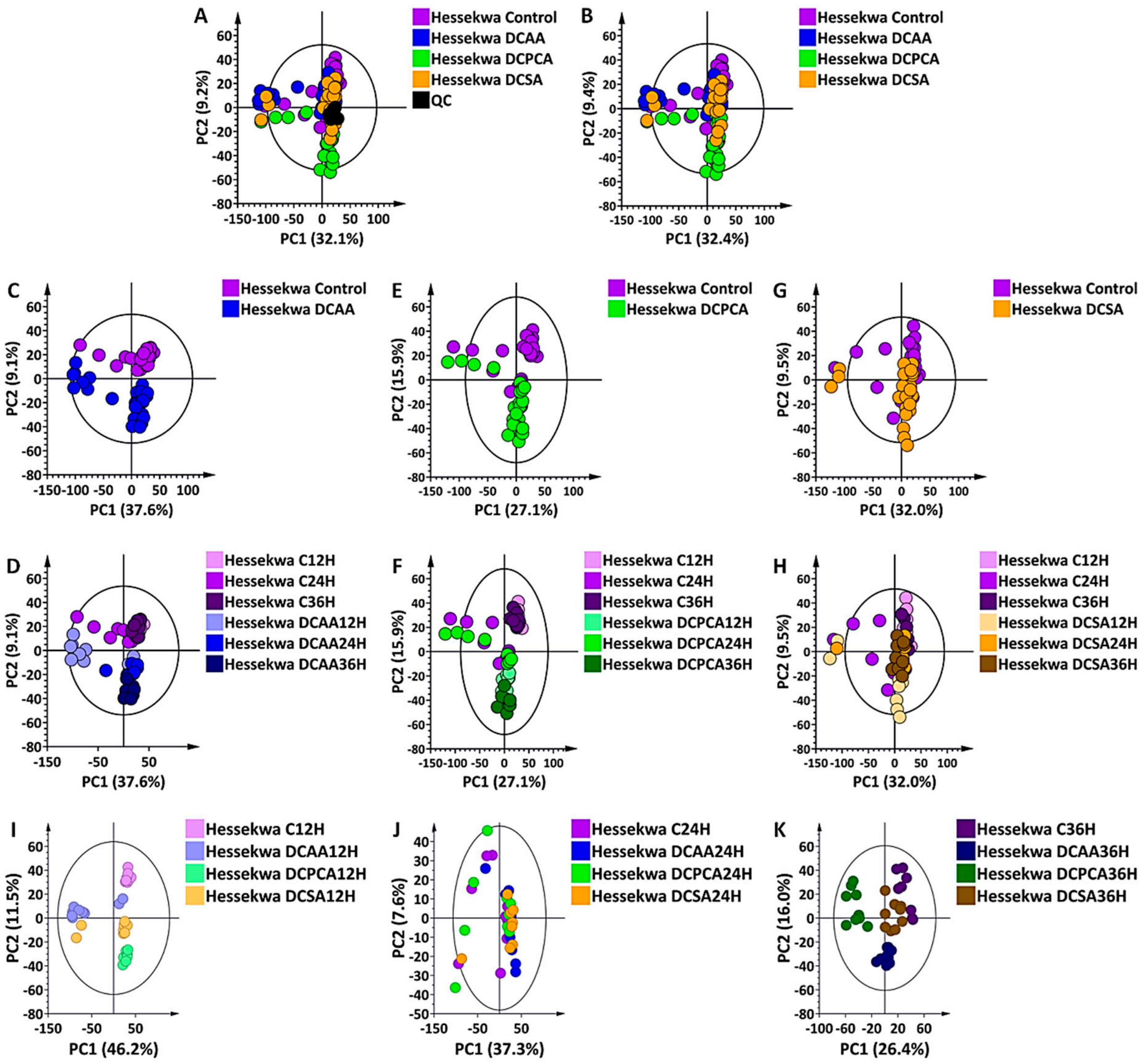

Figure S4. Principal component analysis (PCA) score plot models of ESI(+) data from shoot extracts of the 'Hessekwa' cultivar of *Hordeum vulgare*. All data were UV scaled and the calculated Hoteling's T^2 with a 95% confidence interval is represented by the ellipses present in each PCA model. **A**: 12-component model of all conditions including QCs, explaining 71.6% variation and predicting 56.3% variation; **B**: 12-component model of all conditions explaining 72.3% variation and predicting 56.6% variation; **C**: 7-component model of 3,5-DCAA treated and non-treated samples at 12 h, 24 h and 36 h, explaining 69.7% variation and predicting 53.1% variation; **D**: same as (**C**) but coloured based on time points; **E**: 7-component model of 2,6-DCP-4-CA treated and non-treated samples, explaining 68.6% variation and predicting 48.1% variation; **F**: same as (**E**) but coloured based on time points. **G**: 7-component model of 3,5-DCSA treated and non-treated sample, explaining 66.9% variation and predicting 46.1% variation; **H**: same as (**G**) but coloured based on time points. **I**: 3-component model of all treated and non-treated samples at 12 h, explaining 64.7% variation and predicting 56.2% variation; **J**: 5-component model of all treated and non-treated samples at 24 h, explaining 64.9% variation and predicting 41.4% variation; **K**: 5-component model of all treated and non-treated samples at 36 h, explaining 63.6% variation and predicting 43.2% variation.

(The corresponding set of diagrams for ESI (–) data are presented as Figure 3 in the main text).

Table S1. Metabolic pathways generated from Metabolomics Pathway Analysis (MetPA) in MetaboAnalyst 5.0 and involving selected annotated metabolites. Blue: 3,5-DCAA; Green: 2,6-DCP-4-CA and Orange: 3,5-DCSA.

	**3,5-DCAA**		**2,6-DCP-4-CA**		**3,5-DCSA**	
**Pathway Name**	***p-value***	**Impact**	***p-value***	**Impact**	***p-value***	**Impact**
Phenylpropanoid biosynthesis	1.40E-04	0.070	0.010	0	1.40E-04	0.058
Aminoacyl-tRNA biosynthesis	5.27E-04	0	0.002	0	5.27E-04	0
alpha-Linolenic acid metabolism	6.29E-04	0.337	0.005	0.230	0.007	0.230
Citrate cycle (TCA cycle)	0.003	0.193	0.002	0.193	0.003	0.193
Phenylalanine, tyrosine, tryptophan synthesis	0.004	0.021	0.002	0.021	0.004	0.021
Tryptophan metabolism	0.004	0.241	0.003	0.241	0.004	0.241
Glyoxylate and dicarboxylate metabolism	0.009	0.168	0.006	0.168	0.009	0.168
Biosynthesis of secondary metabolites	●	●	●	●	0.076	1
Isoquinoline alkaloid biosynthesis	0.090	0.411	0.078	0.411	0.090	0.411
Ubiquinone and other terpenoid-quinone biosynthesis	●	●	0.383	0	0.102	0.001
Tropane, piperidine and pyridine alkaloids	0.119	0	0.103	0	0.119	0
Phenylalanine metabolism	0.173	0.423	0.151	0.423	0.173	0.423
Stilbenoid, diarylheptanoid and gingerol	0.160	0.135	●	●	●	●
Flavone and flavonol biosynthesis	0.173	0	●	●	0.173	0
Tyrosine metabolism	0.248	0.167	0.218	0.167	0.248	0.167
Carbon fixation in photosynthetic organisms	0.284	0.058	0.250	0.058	0.284	0.058
Glycerolipid metabolism	0.284	0.117	0.250	0.117	0.284	0.117
Valine, leucine and isoleucine biosynthesis	0.295	0	0.260	0	0.295	0
Biosynthesis of unsaturated fatty acids	0.295	0	0.260	0	0.295	0
Pyruvate metabolism	0.295	0.154	0.260	0.154	0.295	0.154
Alanine, aspartate and glutamate metabolism	0.295	0			0.295	0
Galactose metabolism	0.349	0	0.310	0	0.349	0
Cyanoamino acid metabolism	0.339	0			0.339	0
Glycine, serine and threonine metabolism	0.410	0	0.365	0	0.410	0
Ubiquinone and other terpenoid-quinone	0.428	0	0.383	0		
Valine, leucine and isoleucine degradation	0.447	0	0.400	0	0.447	0
Flavonoid biosynthesis	0.530	0.028	●	●	●	●

(●) absence of the pathway in the corresponding condition.

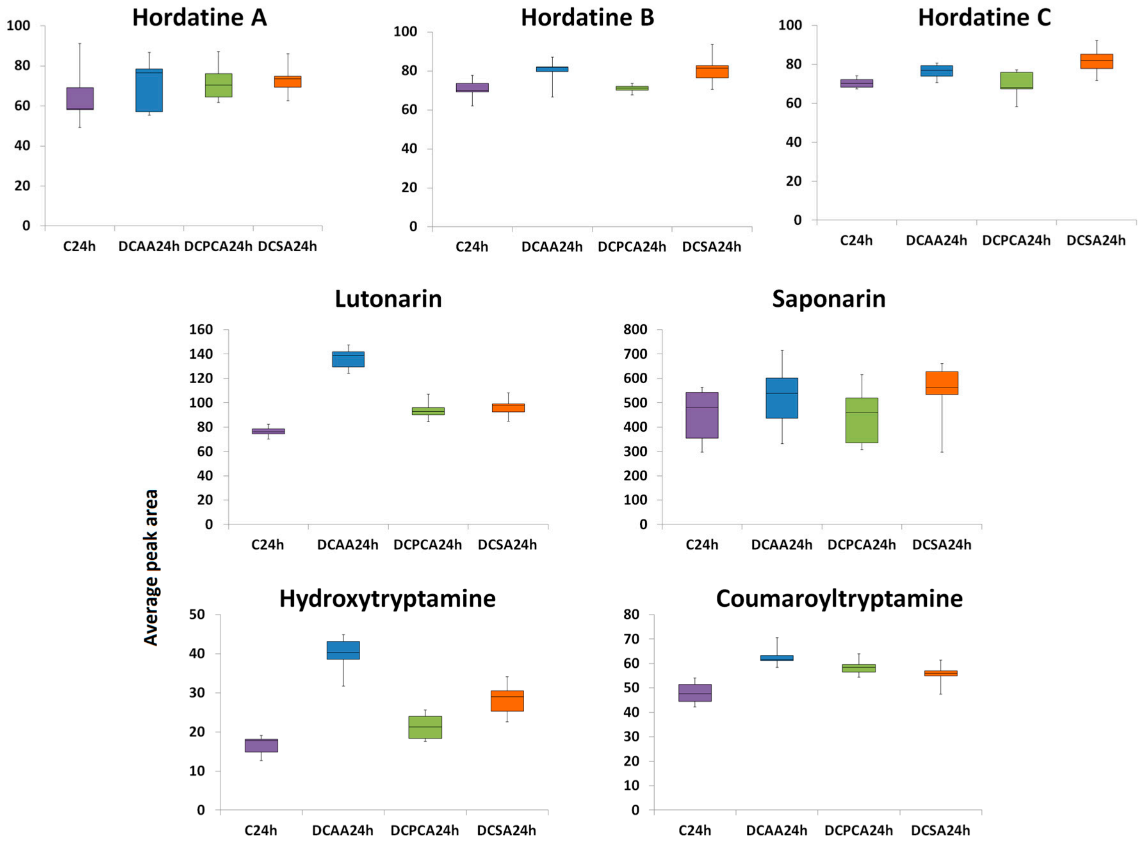

Figure S5. Relative quantification (based on average peak area values) of selected primed / induced metabolites at 24 h. Hordatine A, B and C; flavones lutonarin, saponarin and alkaloids hydroxytryptamine and coumaroyltryptamine.
